# Differences Due to Sex and Sweetener on the Bioavailability of (Poly)phenols in Urine Samples: A Machine Learning Approach

**DOI:** 10.3390/metabo13050653

**Published:** 2023-05-11

**Authors:** Diego Hernández-Prieto, Alberto Garre, Vicente Agulló, Cristina García-Viguera, Jose A. Egea

**Affiliations:** 1 Lab Fitoquimica y Alimentos Saludables (LabFAS), Department of Food Science and Technology (CEBAS-CSIC), Campus Universitario Espinardo, 25, 30100 Murcia, Spain; dprieto@cebas.csic.es; 2 Agronomic Engineering Department, Universidad Politécnica de Cartagena (UPCT), Paseo Alfonso XIII, 48, 30203 Cartagena, Spain; alberto.garre@upct.es; 3 Associated Unit of R&D and Innovation CEBAS-CSIC+UPCT on “Quality and Risk Assessment of Foods”, CEBAS-CSIC, Campus Universitario de Espinardo, 25, 30100 Murcia, Spain; 4 Human Nutrition Unit, Department of Food & Drug, University of Parma, 43125 Parma, Italy; vicente.agullogarcia@unipr.it; 5 Group of Fruit Breeding, Department of Plant Breeding, CEBAS-CSIC, Campus Universitario de Espinardo, 25, 30100 Murcia, Spain; jaegea@cebas.csic.es

**Keywords:** longitudinal trial, biostatistics, clustering, maqui, citrus, beverage, sweeteners, health

## Abstract

Metabolic diseases have been related to the overdrinking of high-sugar content beverages. As a result, the demand for alternative formulations based on plant-based ingredients with health-promoting properties has increased during the last few years. Nonetheless, the design and production of effective formulations requires understanding the bioavailability of these compounds. For this purpose, a two-month longitudinal trial with 140 volunteers was conducted to measure the beneficial effects of a maqui–citrus beverage, rich in (poly)phenols. From data obtained by quantifying metabolites present in urine samples, biostatistical and machine learning (data imputation, feature selection, and clustering) methods were applied to assess whether a volunteer’s sex and the sweetener added to the beverage (sucrose, sucralose, or stevia) affected the bioavailability of (poly)phenol metabolites. Several metabolites have been described as being differentially influenced: 3,4-dihydroxyphenylacetic acid and naringenin with its derivatives were positively influenced by stevia and men, while eriodictyol sulfate and homoeridictyol glucunoride concentrations were enhanced with stevia and women. By examining groups of volunteers created by clustering analysis, patterns in metabolites’ bioavailability distribution as a function of sex and/or sweeteners (or even due to an uncontrolled factor) were also discovered. These results underline the potential of stevia as a (poly)phenol bioavailability enhancer. Furthermore, they also evidence sex affects the bioavailability of (poly)phenols, pointing at a sex-dependent metabolic pathway regulation.

## 1. Introduction

Consumption of high-sugar content beverages is a common habit in developed countries, despite being related to public health issues, such as overweight, obesity, diabetes mellitus II, and cardiovascular diseases [1,2]. However, a new trend has emerged in the designing of plant-based healthy drinks including so-called “superfoods”, such as kale [3], goji berry [4], and maqui berry [5]. Given these two facts, the formulation, production, and analysis of these types of beverages and their health improvements are of great interest [6]. One of the most notable properties of these beverages is their richness in bioactive compounds, particularly (poly)phenols, which are known for their health benefits, such as antioxidant, antimicrobial, anti-inflammatory, anticancer, and cardiovascular protection activities [7].

Recent studies on a maqui–citrus beverage have revealed a complex relationship between the bioavailability of (poly)phenols and the formulation [8,9,10]. In particular, these studies have highlighted the effect of the sweetener present in the drink on the bioavailability, as well as the impact of the consumer’s sex. These studies were based on a longitudinal interventional trial, conducted by the Phytochemistry and Healthy Food Lab (LabFAS), from CEBAS-CSIC, Murcia, where 138 overweight volunteers were provided with fresh drinks sweetened with sucrose, sucralose, or stevia, to be consumed every day for two months. Plasma and urine samples were collected before the first consumption and at the end point of the trial. (Poly)phenols were then identified and quantified using mass spectrometry, leading to several conclusions about maqui and citrus as a source of (poly)phenols, as well as their bioavailability.

To gain deeper insights into this issue through advanced biostatistical methods, our group previously carried out a study that focused on data from plasma samples [9]. The present paper extends this analysis to urine samples to further elucidate the differential metabolism of flavonoids between both sexes with these biostatistical methods.

Research on bioavailability is crucial to understanding (i) how the starting phenolic compounds from maqui (mainly anthocyanins) and citrus (mainly flavanones) are absorbed by the human regulatory system, (ii) how they are metabolized and thus modified by the human physiology, and (iii) how these bioactive metabolites act inside the human body. Thus, the possible health effects provided by the beverages can be measured in terms of the (poly)phenols metabolites present in urine. Additionally, determining the diversity in bioavailability due to the consumer’s sex is necessary to develop a comprehensive understanding of how the nutrients are introduced in diverse human physiologies. Historically, nutritional and clinical trials have not considered sex as a relevant feature [11,12], leading to potentially biased or incomplete results.

The complexity of these questions has made data science an essential tool to answer the questions posed by modern food technology [13,14,15]. Countless measurements are being collected by research and industry laboratories, generating a vast quantity of datasets that often remain unexplored. Using analytical tools from data science, fascinating outcomes can be achieved: from quantifying relationships between environmental, clinical, and industrial factors (discovering patterns and groupings hidden in data) to robustly simulating a dietary intervention. This knowledge would allow for predictions regarding the (beneficial) effect that a food product may have on a variety of consumers.

This paper aims to deploy an innovative data analysis procedure on the data obtained from the longitudinal trial to statistically reinforce the previous results and obtain new insights into them. Specifically, this paper seeks to (i) determine any disparities in bioavailability due to the consumer’s sex, with or without the sweetener, (ii) enhance knowledge about the effects of the sweeteners added to the beverage on the number of metabolites found in urine, and (iii) complement the previous results in plasma with urine data, providing a comprehensive explanation of the observed phenomena. The pursuit of aims (i) and (ii) started in previously cited works, but this paper improves the accuracy of their conclusions through the use of innovative biostatistical and machine learning procedures, whose application to this type of data is certainly innovative. The third aim, (iii), expands the descriptive analysis of the data to provide a complete picture of the issue, including an accurate description of the main effects of the beverage including the variability of additional factors, as well as a thorough examination of all collected samples.

## 2. Materials and Methods

### 2.1. Experimental Phase

The longitudinal study, reported previously by Agulló et al. [16], consisted of an intervention with 138 overweight individuals, who ingested fresh beverages (maqui–citrus based) every day for 2 months. Urine samples were collected at the beginning and at the end of the intake period. Then, phenolic metabolites present in urine samples were identified and quantified by UHPLC-ESI-QqQ-MS/MS, following the method applied and detailed by Agulló et al. [17,18]. The resulting phenolic metabolites were classified as flavanone-derived metabolites and other (poly)phenolic metabolites, named in the work as just “(poly)phenols metabolites”.

### 2.2. Computational Phase

The steps described here, comprising the computational phase, were performed using R Statistical Software v4.1.1 [19].

#### 2.2.1. Dataset

The dataset analyzed includes, for each volunteer: (i) an identification key, (ii) the concentration of metabolites from flavanones and other families of (poly)phenols (such as anthocyanins) present in the urine, (iii) the time of collection of the urine samples (before or after the consumption of the beverage), (iv) the sweetener added to the beverage, and (v) the sex of the consumer. The factors (and interactions) included in the study were time (independent consumption of the beverage regardless of the other factors), sex–time (effect of the beverage on the different metabolism of both sexes), sweetener–time (how the sweetener added to the beverage affects the concentration of metabolites), and sex–sweetener–-time (how a particular sweetener added to the beverage impacts the metabolism of the different sexes).

#### 2.2.2. Main Pipeline

The computational methodology followed in this work can be found in Hernández-Prieto et al. [9]. Briefly, it consisted of the following steps:

1. Preprocessing: normalization of data, descriptive statistics, and evaluation to check the suitability of the techniques applied afterward, presented in supplementary material (Table S2);
2. Three-way paired ANOVA followed by multiple pairwise *t*-tests: this type of ANOVA calculates the effect of three factors (time, sex, and sweetener) on the mean value of a continuous variable (metabolite concentration), with no independence between groups, i.e., the groups correspond to different sampling times. The *t*-test compares pairwise every level of the factors to endorse ANOVA and obtain more information about the relationship between levels of factors. By plotting the data in a boxplot, the direction and magnitude of the factor effect over the different groups can be visualized;
3. Data imputation: to perform feature selection and clustering analysis, all datasets were improved by multivariate data imputation techniques that fill the empty spaces using regression algorithms;
4. Feature selection: for improving clustering performance, Boruta’s algorithm for feature selection [20] is applied, choosing the most important/significant variables. The selected variables are listed in Results, Section 3.2.1;
5. Clustering: to describe interesting groups and search for patterns in data, clustering analysis is implemented. To tune clustering performance, the number of clusters and clustering technique are chosen via the R packages “NbClust” [21] and “clValid” [22]. The chosen parameters are compiled in Results, Section 3.2. To visualize the cluster distribution and the contribution of every variable to the cluster composition, a corresponding biplot has been charted.

## 3. Results

### 3.1. Effects of Experiment Factors over the Metabolite Concentration Values after Beverage Intake through ANoVa Technique

#### 3.1.1. (Poly)phenol Metabolites Set

The characterization of the (poly)phenolic content of the beverages, by individual anthocyanins and flavanones, and its metabolites once consumed, is detailed in Agulló et al. [8]. The full list of metabolites identified and quantified from this set were Caffeic Acid (CA), CA-Glucuronide (CA-G), CA-Glucuronide-Sulfate (CA-GS), the total amount of CA and its derivatives (Total CA), 3,4-Dihydroxyphenylacetic acid (DHPAA), DHPAA-Glucuronide (DHPAA-G), DHPAA-Glucuronide-Sulfate (DHPAA-GS), DHPAA-Di-Sulfate (DHPAA-SS), the total amount of DHPAA and its derivatives (Total DHPAA), *trans* Ferulic Acid-Glucuronide (TFA-G), *trans* Ferulic Acid-Sulfate (TFA-S), the total amount of TFA and its derivatives (Total TFA), Vanillic Acid (VA), VA-Di-Glucuronide (VA-GG) and VA-Di-Sulfate (VA-SS), VA-Glucuronide-Sulfate (VA-GS), and the total amount of VA and its derivatives (Total VA).

**Figure 1:**
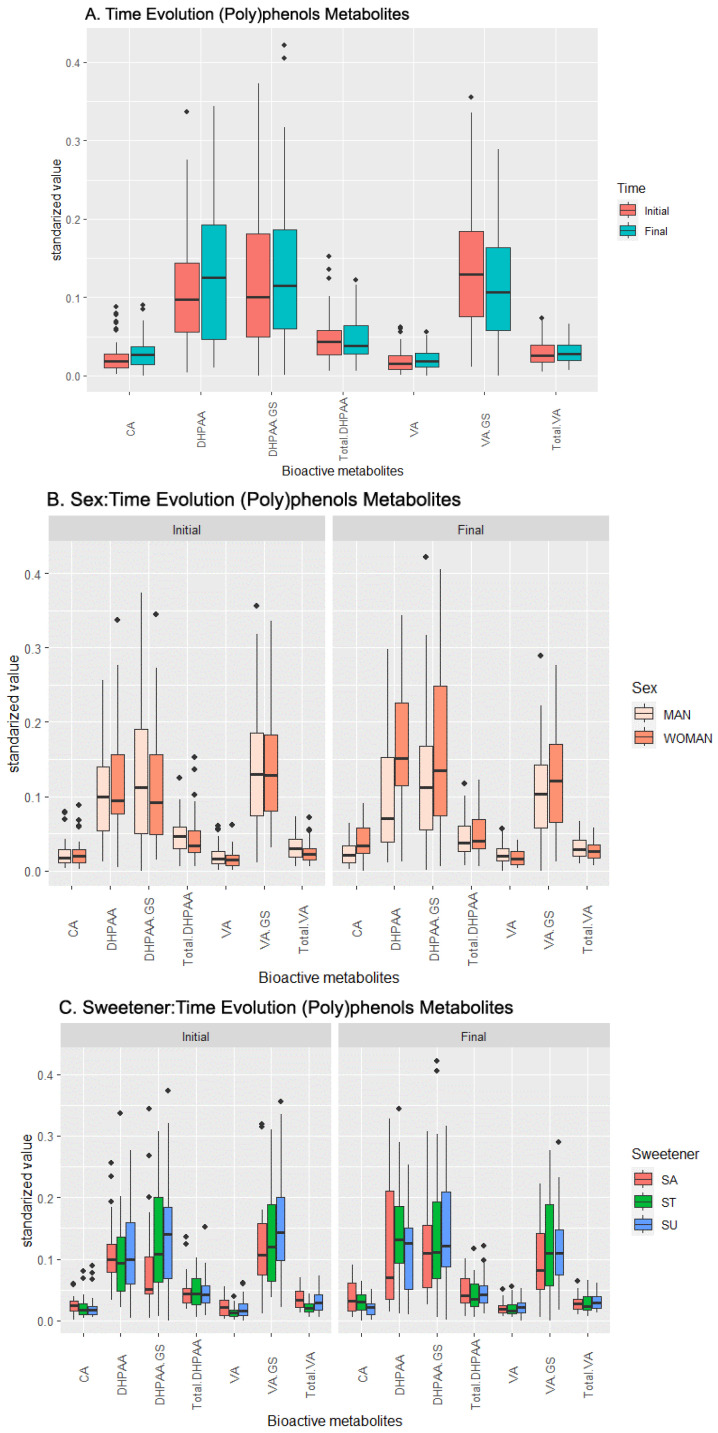
Temporal evolution of the effects of the beverage over the response (metabolic values) in the (poly)phenol metabolites set. (**A**) Effect of the beverage over time, (**B**) effect of the interaction sex–time, (**C**) effect of the interaction sweetener–time. Abbreviations: Caffeic Acid (CA), 3,4-Dihydroxyphenylacetic acid (DHPAA), DHPAA-Glucuronide-Sulfate (DHPAA-GS), total amount of DHPAA and its derivatives (Total DHPAA), Vanillic Acid (VA), VA-Glucuronide-Sulfate (VA-GS), and total amount of VA and its derivatives (Total VA).

The analysis of variance reveals an influence of time (which translates into the effect of the beverage over the concentration of metabolites independently of other factors) over DHPAA, VA, VA-GS, and Total VA, as seen in Table 1 and Figure 1A. DHPAA presented the highest increase in its concentration with time, while VA-GS was the only compound that displayed a decrease in its concentration in urine. The data show that the sex affects the temporal variation in the concentrations of CA and DHPAA (Figure 1B). For both metabolites, there is a higher increase in women than in men, to the point that DHPAA even decreases in men. Finally, the added sweetener only modulates DHPAA and its derivative DHPAA-GS, increasing its concentration (Figure 1C).

The pairwise *t*-test supports the ANOVA results (complete in the supplementary material, Table S2), but also reveals few relevant effects and many non-relevant interactions: the weakest (but significant) levels are spotted in the CA concentration, which is positively related with females who consumed the beverage with sucrose added; in the Total DHPAA, whose concentration increases with females and sucralose interaction; and in the VA-GS, whose concentration is negatively influenced by beverages with sucrose added consumed by females. On the other hand, the analysis identified a strong effect on the DHPAA concentration in males whose beverage had been sweetened with stevia, and a lower effect on females, regardless of the sweetener, and in females whose beverage had stevia added.

**Table 1:** *p*-value for the ANOVA and pairwise *t*-test analysis of (poly)phenol metabolites data results. In bold, those *p*-values < 0.05 from ANOVA. In the *t*-test results, (+) means a positive relation and (-) a negative relation. More than one symbol indicates a higher grade of relation, based on a *p*-value < 0.01 for (++)/and *p*-value < 0.001 for (+++). CA-G, CA-GS, Total CA, DHPAA-G, DHPAA-SS, TFA-G, TFA-S, Total TFA, VA-GG, and VA-SS were discarded due to non-significance in both ANOVA and *t*-test. Abbreviations: Male (M), Female (F), Sucrose (SA), Sucralose (SU), Stevia (ST), Time (T), non-relevant (NR), Caffeic Acid (CA), CA-G Glucuronide (CA-G), CA-Glucuronide-Sulfate (CA-GS), total amount of CA and its derivatives (Total CA), 3,4-Dihydroxyphenylacetic acid (DHPAA), DHPAA-Glucuronide (DHPAA-G), DHPAA-Glucuronide-Sulfate (DHPAA-GS), DHPAA-Di-Sulfate (DHPAA-SS), total amount of DHPAA and its derivatives (Total DHPAA), *trans* Ferulic Acid-Glucuronide (TFA-G), *trans* Ferulic Acid-Sulfate (TFA-S), total amount of TFA and its derivatives (Total TFA), Vanillic Acid (VA), VA-Di-Glucuronide (VA-GG) and VA-Di-Sulfate (VA-SS), VA-Glucuronide-Sulfate (VA-GS), total amount of VA and its derivatives (Total VA).

Compounds	Factors and Interactions of Factors (*p*-Value)			
	Time	Sex–Time	Sweetener–Time	Pairwise *t*-Test
CA *	9.13 × 10^−1^	**2.40 × 10^−2^**	8.45 × 10^−1^	F/SA(+),F(+)
DHPAA	**2.00 × 10^−3^**	**4.30 × 10^−3^**	**2.60 × 10^−3^**	ST/M(+++), ST/F(+), ST(++), F(+), T(++)
DHPAA-GS *	6.34 × 10^−1^	1.52 × 10^−1^	**3.00 × 10^−2^**	NR
Total DHPAA	1.55 × 10^−1^	1.92 × 10^−1^	6.68 × 10^−1^	F/SU(+)
VA	**2.50 × 10^−2^**	9.70 × 10^−2^	5.50 × 10^−1^	NR
VA-GS	**2.80 × 10^−2^**	9.53 × 10^−1^	2.73 × 10^−1^	F/SA(-), SA(-), T(-)
Total VA	**4.70 × 10^−2^**	1.08 × 10^−1^	5.94 × 10^−1^	NR

* More than 20 individuals were removed by the algorithm for containing missing values.

#### 3.1.2. Flavanone Metabolites Set

The flavanone compounds found in the beverages and their metabolites once consumed are detailed in Agulló et al. [8]. In this case, only phase II metabolites derived exclusively from flavanones were considered. The compounds identified and quantified in the urine samples were Eriodictyol (E), E-Glucuronide (EG), E-Sulfate (ES), the total amount of E and its derivatives (Total E), Homoeriodyctiol (HE), HE-Glucuronide (HE-G), HE-Di-Glucuronide (HE-GG), the total amount of HE and its derivatives (Total HE), Naringenin (N), N-Glucuronide (NG), N-Di-Glucuronide (NGG) N-Sulfate (NS), and the total amount of Naringenin (Total N).

**Figure 2:**
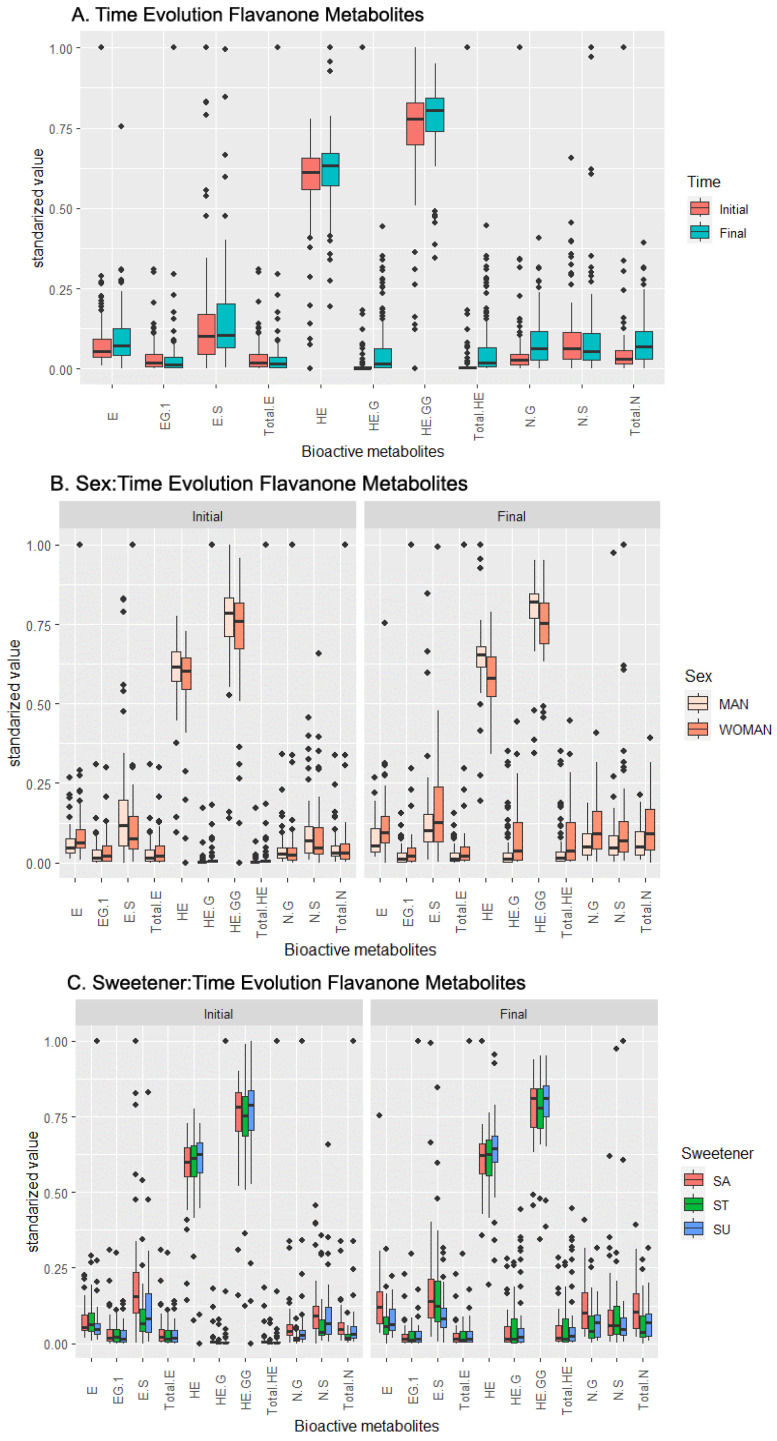
Temporal evolution of the effects of the beverage over the response (metabolic values) in the flavanone metabolites set. (**A**) Effect of the beverage over time, (**B**) effect of the interaction sex–time, (**C**) effect of the interaction sweetener–time. Abbreviations: Eriodictyol (E), E-Glucuronide (EG), E-Sulfate (ES), total amount of E and its derivatives (Total E), Homoeriodyctiol (HE), HE-Glucuronide (HE-G), HE-Di-Glucuronide (HE-GG), total amount of HE and its derivatives (Total HE), Naringenin-Glucuronide (NG), Naringenin-Sulfate (NS), total amount of Naringenin (Total N).

Based on this dataset, the ANOVA concludes that the concentrations of the following metabolites are influenced by the independent effect of time: HE, HE-G, HE-GG, NG, Total HE, and Total N (Table 2). The analysis of Figure 2A shows an increase in the concentration of every single metabolite with time, with the highest increases observed in HE-G and Total HE.

Meanwhile, ES is the only compound influenced by the sex factor, based on the ANOVA, with its concentration increasing in women (Figure 2B). According to the pairwise *t*-test and boxplot chart in Figure 2B, HE and HE-GG exhibited increased concentrations in men. For the rest of the compounds affected by the sex factor, the boxplot shows only small shifts in their concentrations.

Examining the sweetener factor, the ANOVA reveals a sweetener influence over the E and ES concentrations. According to Figure 2C and the pairwise *t*-test (supplementary material, Table S3), the E concentration was influenced mainly by sucrose, and the ES concentration was influenced mainly by stevia, increasing their concentrations. In a lesser magnitude, HE-G and Total HE increased their concentration when stevia was added to the product. The stevia regulation seemed to be the most impactful among all the sweeteners included in the study. The pairwise *t*-test provided additional insight into the interaction between the sex and sweetener, which was among the most relevant: the positive influence of stevia on females was observed for the ES, HE-G, and Total HE concentrations, while the same positive influence was observed in the stevia-sweetened beverages for males in the NG and Total N concentrations (Table 2).

**Table 2:** *p*-value for the ANOVA and pairwise *t*-test analysis of flavanone metabolites results. In bold, those *p*-values < 0.05 from ANOVA. In the *t*-test results, (+) means a positive relation and (-) a negative relation. More than one symbol indicates a higher grade of relation, based on a *p*-value < 0.01 for (++)/ and *p*-value < 0.001 for (+++)/. N and NGG were discarded due to non-significance in both ANOVA and *t*-test. Abbreviations: Male (M), Female (F), Sucrose (SA), Sucralose (SU), Stevia (ST), Time (T), Eriodictyol (E), E-Glucuronide (EG), E-Sulfate (ES), total amount of E and its derivatives (Total E), Homoeriodyctiol (HE), HE-Glucuronide (HE-G), HE-Di-Glucuronide (HE-GG), total amount of HE and its derivatives (Total HE), Naringenin-Glucuronide (NG), Naringenin-Sulfate (NS), total amount of Naringenin (Total N).

Compounds	Factors and Interactions (*p*-Value)			
	Time	Sex–Time	Sweetener–Time	Pairwise *t*-Test
E	2.44 × 10^−1^	9.00 × 10^−1^	**2.70 × 10^−2^**	F/SA(+), SA(++)
EG	7.83 × 10^−1^	2.67 × 10^−1^	2.30 × 10^−1^	M/SA(+)
ES	5.42 × 10^−1^	**1.20 × 10^−2^**	**2.80 × 10^−2^**	F/SA(+), F/ST(+), F(++), ST(+)
Total E	7.61 × 10^−1^	2.48 × 10^−1^	2.28 × 10^−1^	M/SA(+)
HE	**1.30 × 10^−2^**	2.28 × 10^−1^	8.89 × 10^−1^	M/SA(+), M/SU(+), M(++), SU(+). T(++)
HE-G	**1.00 × 10^−3^**	4.76 × 10^−1^	7.00 × 10^−1^	M/SA(+), M/ST(+), F/ST(++), M(+++), SA(+), ST(+++), T(++)
HE-GG	**8.00 × 10^−3^**	8.10 × 10^−1^	9.03 × 10^−1^	M(+), T(++)
Total HE	**6.66 × 10^−4^**	5.73 × 10^−1^	7.65 × 10^−1^	M/SA(+), M/ST(+), M/SU(+), F/ST(++), M(+++), F(+), SA(+), ST(+++), T(+++)
NG	**2.00 × 10^−3^**	2.30 × 10^−1^	1.83 × 10^−1^	M/SA(+), M/ST(+++), F/SA(+), F/ST(+), M(+), F(+). SA(++), ST(+++), T(++)
NS	6.45 × 10^−1^	8.70 × 10^−2^	2.47 × 10^−1^	M/SA(+), M/SU(+)
Total N	**3.00 × 10^−3^**	2.21 × 10^−1^	2.00 × 10^−1^	M/ST(+++), F/SA(+), F/ST(+), M(+), F(+), SA(++), ST(+++), T(++)

### 3.2. Patterns and Groups of Interest Extracted from Experimental Data by Clustering Analysis Technique

For every dataset, the chosen algorithm technique to perform clustering was PAM, supported by clValid package [22], and the number of clusters selected was six, by the package NbClust [21]. The distribution of values of the concentrations of metabolites for every cluster are in the supplementary material, Table S4.

#### 3.2.1. Selection of Most Descriptive Metabolites to Improve Clustering Analysis Performance

Results for feature selection using the Boruta algorithm are reported in Table 3.

#### 3.2.2. (Poly)phenol Metabolites Set

**Figure 3:**
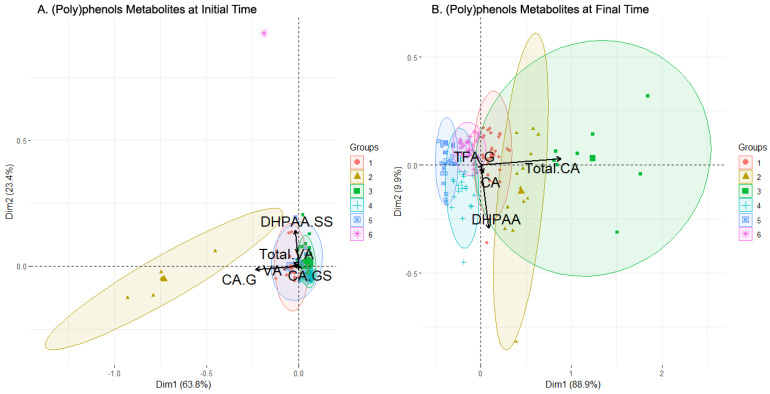
Biplot of the clusters generated by clustering analysis applied (poly)phenol metabolites set of data before (**A**) and after (**B**) consumption of the beverage. In black color, the arrows with the contribution to the composition of the clusters of the variables. Abbreviations: Caffeic Acid (CA), CA-Glucuronide (CA-G), CA-Glucuronide-Sulfate (CA-GS), 3,4-Dihydroxyphenylacetic acid (DHPAA), DHPAA-Di-Sulfate (DHPAA-SS), *trans* Ferulic Acid-Glucuronide (TFA-G), Vanillic Acid (VA), total amount of VA and its derivatives (Total VA).

Based on the data analyzed for the first subset of ((Poly)phenol metabolites) using cluster analysis, at the initial time, six clusters were identified (Table 4 for summary of composition, Figure 3A for PCA projection). Clusters 1, 2, 3, and 5 had largely overlapping and highly concentrated individuals in the PCA projection, while cluster 2 had few and widely scattered individuals. Cluster 6 was mono-individual and very separated from the other data points in the PCA. The most populated clusters were clusters 3 (20 men and 21 women) and 4 (29 men and 15 women). The loadings associated with the variables ((poly)phenol metabolites present in the urine) selected by the feature selection algorithm (Table 3) are shown in Figure 3A as arrows. CA-G was found to be the main component of cluster 2 and partially of cluster 5, with a single direction and a large length. The rest of the metabolites followed the same direction, mainly composing clusters 1 and 4, and again partially of cluster 5, with DHPAA-SS as the most determinant. Cluster 3 was not determined by any of the selected features and cluster 6 is too far away in projection to associate it with any feature.

**Figure 4:**
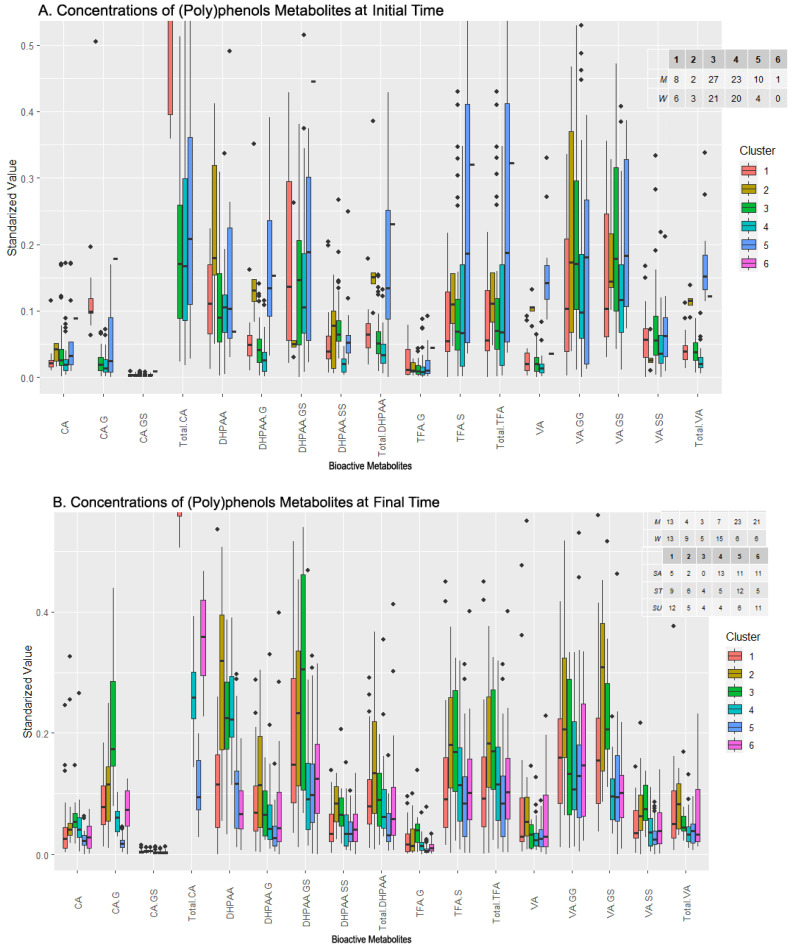
Levels of bioactive (poly)phenol metabolites within the clusters generated by clustering analysis before (**A**) and after (**B**) beverage consumption and composition of clusters in terms of sexes and sweeteners added to the drink. Abbreviations: Caffeic Acid (CA), CA-Glucuronide (CA-G), CA-Glucuronide-Sulfate (CA-GS), total amount of CA and its derivatives (Total CA), 3,4-Dihydroxyphenylacetic acid (DHPAA), DHPAA-Glucuronide (DHPAA-G), DHPAA-Glucuronide-Sulfate (DHPAA-GS), DHPAA-Di-Sulfate (DHPAA-SS), total amount of DHPAA and its derivatives (Total DHPAA), *trans* Ferulic Acid-Glucuronide (TFA-G), *trans* Ferulic Acid-Sulfate (TFA-S), total amount of TFA and its derivatives (Total TFA), Vanillic Acid (VA), VA-Di-Glucuronide (VA-GG) and VA-Di-Sulfate (VA-SS), VA-Glucuronide-Sulfate (VA-GS), total amount of VA and its derivatives (Total VA).

By examining the levels of the different compounds in the clusters for this initial time dataset (Figure 4A), outliers were trimmed for visualization purposes, leaving out some values, especially in clusters 1, 2, and 6. Taking this into account, as well as the number of individuals and males and females in each cluster, relationships of interest can be determined between the sexes and compounds at the initial time in this case. More attention is also paid to variables that have been previously selected (Table 3).

The first cluster was balanced in terms of the sexes and presents stable levels of every compound, with the Total CA being the most elevated level. The third cluster was similar to the first, also balanced in the sexes population, and presented a low concentration level of every metabolite, but is more populated (48 individuals). As expected, by examining the biplot (Figure 3A), cluster 3 presented low concentration levels in the selected features, even being the lowest in almost every metabolite. Cluster 4 was the most populated one, was balanced in the sexes (23 M/20 W), and showed a more complex diversity than clusters 1 and 3. The Total CA level presented was average, contrary to the TFA-G, DHPAA-G, or Total VA levels, where cluster 4 was among the lowest. Cluster 5 was composed of a greater number of men and presented a varied value distribution, with high levels of Total DHPAA, DHPAA-G, DHPAA-GS TFA-S, Total TFA, and Total VA compared to the other clusters, but with wide variance in the values. Finally, clusters 2 and 6 had far fewer individuals (5 and 1, respectively) and their compound levels were usually much lower or were even trimmed off for high values (CA-G for cluster 2, DHPAA-SS for cluster 6). Other trimmed-off variables were, for example, Total CA in both clusters.

At the final sampling time (after the intake), six clusters were identified by cluster analysis (Figure 3B). Two clusters, clusters 2 and 3, were quite scattered with few individuals, while four clusters, clusters 1, 4, 5, and 6, had more individuals, were more concentrated, and partially overlapped each other. The plot illustrates the loadings of the variables selected by the feature selection algorithm (Table 3). Clusters 1, 2, and 3 were highly defined by the Total CA, whose arrow was the second longest. On the other hand, DPHAA defined clusters 1, 4, and 5 with the highest magnitude. TFA-G and CA had a smaller magnitude.

The boxplot chart for the final sampling time (which also summarizes the sex and sweetener composition of clusters, Figure 4B) showed that the composition of the clusters was varied. Clusters 2, 3, and 4 grouped more women than men, while clusters 5 and 6 the opposite. Cluster 1 was completely balanced. On the other hand, the only cluster where one sweetener clearly dominated was cluster 4, with sucrose prevailing over the rest. In the selected variables, it was observed that the levels of clusters 1 and 2 in the Total CA were strongly elevated and were cut by trimming. These clusters had less sucrose presence compared to the rest, which may indicate a positive influence of the alternative sweeteners over the Total CA. There were also more women considering the two clusters, but the difference with the number of men is too low to point to a possible regulation. In contrast, CA had lower levels in these clusters, with clusters 3 and 4 presenting the highest levels. DHPAA obtained strong values from cluster 2 above all and, to a lesser extent, from clusters 3 and 4. This can be translated into a higher bioavailability in women of this metabolite since they were a majority when grouping these three clusters together (2, 3, 4). Cluster 3 was the most prominent in TFA-G, with the level of the rest of the clusters being very low. Clusters 5 and 6, which group the majority of males, always had very low values, except cluster 6 in the Total CA. This fact may relate to an increase in the bioavailability of this metabolite for men when sucrose or sucralose is present in their beverages. Finally, it was seen that clusters 2 and 3 combined contained most of the highest concentration levels of metabolites, as expected since they were the most different regarding the rest of the clusters (as seen in Figure 3B), which seemed concentrated around lower concentration levels overall. These two clusters were characterized by an almost total absence of sucrose and a majority of women, which provided an idea regarding an increase in the (poly)phenols metabolites’ concentration levels in women with alternative sweeteners. Lastly, VA and its derivatives had an enhanced concentration in clusters with more individuals with alternative sweeteners to sucrose added to their drinks, e.g., clusters 1, 2, and 3. In brief, a positive influence of alternative sweeteners over the Total CA concentration could be observed, and another positive influence for women in the DHPAA concentration was stated. Sucrose and sucralose were evidenced to increase the Total CA concentration levels in men by observing cluster 6, and, with alternative sweeteners to sucrose, there was an increase in the bioavailability of metabolites from (poly)phenols in women, as seen in clusters 1, 2, and 3. Finally, levels of VA were enhanced by alternative sweeteners regardless of the sex, also seen in clusters 1, 2, and 3.

Even after the trimming, the loadings of CA-GS remained extremely low, indicating that this compound had little influence on the results of the cluster analysis. The supplementary material provides a boxplot focused on this compound, illustrating how its levels were distributed among the samples (Figure S1). For the initial sampling time, the levels were low and almost identical for the first five clusters, with the top being cluster 6, albeit with very low concentrations. After the clinical trial (final sampling time), the concentrations of CA-GS for every cluster remained at similar values, but with a wider variance. Furthermore, at the initial time, cluster 6 only contained one individual, volunteer 66, who was a woman with high levels of all compounds but DHPAA and VA. At the end time, she became part of cluster 4 because every other participant had an increase in their overall concentration of the compounds after the consumption of the beverage, reaching similar concentrations as individual 66. Her levels are shown in the supplementary material, Table S5.

**Table 4:** Summary of clusters produced by clustering analysis performed on data from (poly)phenol metabolites at initial and end time of trial. Abbreviations: Male (M), Female (F), Sucrose (SA), Sucralose (SU), Stevia (ST), Time (T), Caffeic Acid (CA), CA-Glucuronide (CA-G), CA-Glucuronide-Sulfate (CA-GS), 3,4-Dihydroxyphenylacetic acid (DHPAA), DHPAA-Glucuronide (DHPAA-G), DHPAA-Di-Sulfate (DHPAA-SS), total amount of DHPAA and its derivatives (Total DHPAA), *trans* Ferulic Acid-Glucuronide (TFA-G), *trans* Ferulic Acid-Sulfate (TFA-S), total amount of TFA and its derivatives (Total TFA), Vanillic Acid (VA), total amount of VA and its derivatives (Total VA).

(Poly)phenol Metabolites at Initial Time (day 0)																		
Cluster	1			2			3			4			5			6		
Sex #	M: 8		F: 6	M: 2		F: 3	M: 27		F: 21	M: 23		F: 10	M: 10		F: 4	M: 1		F: 0
High values	DHPAA-SS			DHPAA-SS, CA-G, Total CA						Total CA			DHPAA, Total DHPAA, TFA-S, Total TFA, Total VA					
Low values				CA, CA-GS						TFA-G, DHPAA-G, Total VA			CA-G, DHPAA-SS					
**(Poly)phenol Metabolites at Final Time (day 60)**																		
Cluster	1			2			3			4			5			6		
Sex #	M: 13		F: 13	M: 4		F: 9	M: 3		F: 5	M: 7		F: 15	M: 23		F: 6	M: 21		F: 6
Sweetener # SA/ST/SU	5	9	12	2	6	5	0	4	4	13	5	4	11	12	6	11	5	11
High values	Total CA			Total CA, DHPAA			CA, DHPAA, TFA-G			CA, DHPAA						Total CA		
Low values	CA			CA									All			All but Total CA		

#### 3.2.3. Flavanone Metabolites Set

**Figure 5:**
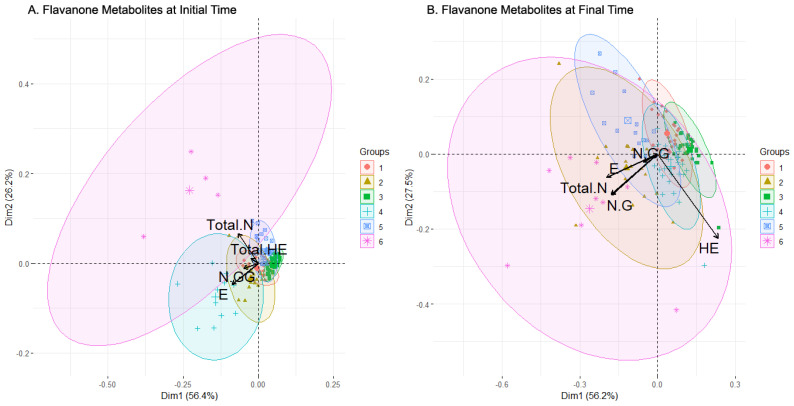
Biplot of the clusters generated by clustering analysis applied to flavanone metabolites set of data before (**A**) and after (**B**) consumption of the beverage. In black color, the arrows with the contribution to the composition of the clusters of the variables. Abbreviations: Eriodictyol (E), Homoeriodyctiol (HE), total amount of HE and its derivatives (Total HE), Naringenin-Glucuronide (N-G), Naringenin-Di-Glucuronide (N-GG), total amount of Naringenin (Total N).

The PCA projection of the clusters for the flavanone metabolites subset at the initial sampling time revealed six clusters (Table 5 for summary of composition, Figure 5A for PCA projection). Four of these clusters (clusters 1, 2, 3, and 5) were clearly concentrated and slightly overlapped, while another cluster (cluster 4) was more dispersed with fewer individuals (cluster 4), and a marginal cluster (cluster 6) contained only four highly dispersed individuals. The feature selection method selected E, Total HE, N-GG, and Total N as relevant metabolites (Table 3), with their loadings illustrated by arrows in the plot. Clusters 2 and 4 were defined by the large magnitude of the loading associated with E, and, together with cluster 1, by the relevant but smaller magnitude of the N-GG loading. Total N defined the marginal cluster 6 and, partially, cluster 5, while Total HE defined clusters 1, 2, and partially cluster 6.

**Figure 6:**
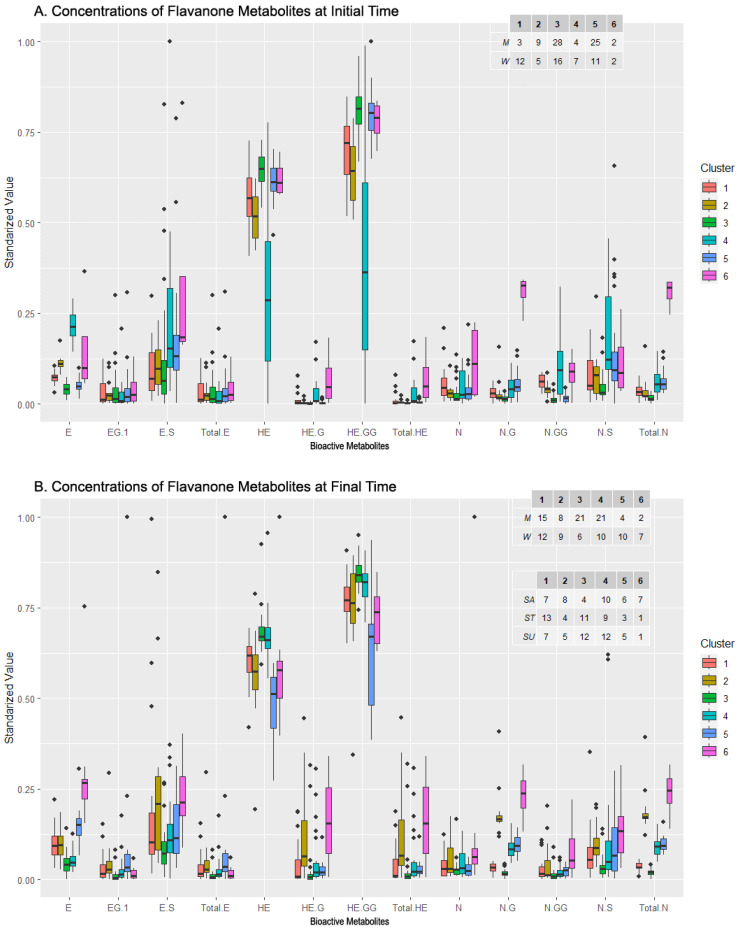
Levels of bioactive Flavanone metabolites from the clusters generated by clustering analysis before (**A**) and after (**B**) beverage consumption and composition of clusters in terms of sexes and sweeteners added to the drink. Abbreviations: Eriodictyol (E), E-Glucuronide (EG), E-Sulfate (ES), total amount of E and its derivatives (Total E), Homoeriodyctiol (HE), HE-Glucuronide (HE-G), HE-Di-Glucuronide (HE-GG), total amount of HE and its derivatives (Total HE), Naringenin (N), N-Glucuronide (N-G), N-Di-Glucuronide (N-GG), N-Sulfate (N-S), total amount of Naringenin (Total N).

The distribution of the concentration levels of the metabolite at the initial time can be seen in Figure 6A. Clusters 1 and 4 contained more women than men and presented lower levels of HE and HE-GG (especially cluster 4) and derivatives as well as higher levels of derivatives of N, in particular, N-G and N-GG. The clusters proportionally more abundant in men were 2, 3, and 5, and they did not seem to show a pattern in their distribution, except for the low levels in N and its derivatives. On a different note, cluster 3, which was close but above cluster 5 in Figure 6A, presented a high level of both HE and HE-GG, but diverged from 5 in ES and NS levels, as cluster 5 presented greater concentrations of these metabolites. Lastly, cluster 6 was balanced in the number of men and women and presented the highest level in several metabolites: ES, N, NG, and Total N. In short, a relationship between women, low levels of E and its derivatives, and high levels of N and its derivatives was observed. In addition, clusters composed mainly of men presented a higher level of HE and HE-GG.

Examining the results of the clustering analysis in the flavanone metabolites’ concentration data after the intake of the beverages, six clusters are observed in the PCA projection (Figure 5B). Three of them (clusters 1, 4, and 5) were partially overlapped, with their individuals concentrated and centered around the origin of coordinates, while another cluster (cluster 3) was apart from those three with a similar shape (cluster 3). Another cluster (cluster 2) was more dispersed but also partially centered (cluster 2), and there was a widely scattered one, so much so that its ellipse almost comprises the rest of the clusters (cluster 6). Boruta’s algorithm for feature selection chose E, HE, N-G, N-GG, and Total N (Table 3). The arrow representing the loadings for HE defined the composition of clusters 3 and 4 strongly, and partially the composition of clusters 1 and 6. The loadings of NG and Total N were practically identical, and similar to the E loading, being the three that were more important in the compositions of clusters 2 and 6. Cluster 5 was undefined by the features selected, except for the small N-GG loading.

The composition of each cluster, along with a comparison between the initial and final sampling times, revealed the relationship between the sex, sweetener, and compound bioavailability. Clusters 1 and 2 were balanced in the sex but not in the sweeteners, with stevia prevailing in the first cluster while sucrose prevailed in the second. In general, cluster 2 displayed a larger metabolite concentration than cluster 1, except for HE and HE-GG. Men were grouped in a more significant number in clusters 3 and 4, whose composition in the sweeteners was balanced, except for the sucrose in cluster 3, where it was much less represented. In the metabolites, both clusters had similar concentration levels (high in HE and HE-GG), except for NG and Total N, where cluster 4 presented higher values. Clusters 5 and 6 had more women than men, and almost no difference between the sweeteners was observed in cluster 5, while cluster 6 contained mainly sucrose-sweetened cases. Both clusters presented high levels of E and ES (just above the average for cluster 5), and low levels of HE and HE-GG, but differed in E-G and Total E, presenting higher concentrations in cluster 5. These clusters also had significantly different levels in ES, HE-G, Total HE, NG, N-GG, and Total N, where cluster 6 showed higher values.

In summary, regardless of sex, sucrose appears to stimulate the bioavailability of flavanones (as seen in clusters 1 and 2). In men, lack of sucrose tends to decrease NG and Total N. In women, regardless of the sweetener, E and ES are elevated whereas HE and HE-G are low. Women who consumed sucrose tended to have low values of E-G and Total E and high values of ES, HE-G, Total HE, NG, and N-GG, as seen in cluster 6.

**Table 5:** Summary of clusters produced by clustering analysis performed on data from flavanone metabolites at initial and final time of trial. Abbreviations: Male (M), Female (F), Sucrose (SA), Sucralose (SU), Stevia (ST), Eriodictyol (E), Homoeriodyctiol (HE), HE-Di-Glucuronide (HE-GG), Naringenin-Glucuronide (NG), Naringenin-Di-Glucuronide (N-GG), total amount of Naringenin (Total N).

Flavanone Metabolites at Initial Time (day 0)																		
Cluster	1				2		3		4			5			6			
Sex #	M: 3		F: 12		M: 9	F: 5	M: 28	F: 16	M: 4		F: 7	M: 25	F: 11		M: 2		F: 2	
High values	N and derivatives						HE, HE-GG		N and derivatives			HE, HE-GG						
Low values	E and derivatives								E and derivatives, HE, HE-GG									
**Metabolites from Flavanones at Final Time (day 60)**																		
Cluster	1				2		3		4			5			6			
Sex #	M: 8		F: 6		M: 2	F: 3	M: 27	F: 21	M: 23		F: 10	M: 10	F: 4		M: 1		F: 0	
Sweetener # SA/SU/ST	7	13	7	8	4	5	4	11	12	10	9	12	6	3	5	7	1	1
High values	HE, HE-GG				HE, HE-GG		HE, HE-GG		N-G, Total N, HE, HE-GG			E and derivatives			E and derivatives, NG, N-GG, and Total N			
Low values							N-G, Total N											

## 4. Discussion

The current study complements the results obtained in the plasma samples presented in Hernández-Prieto et al. [9] with the analysis of the urine samples extracted by Agulló et al. [8]. Although this study applies a similar data-oriented methodology, it provides a broader view of the bioavailability of the health-promoting compounds in the maqui–citrus beverage. Despite using a different approach to estimate the bioavailability (plasma versus urine), both studies conclude that both the consumer’s sex and the interaction with the sweetener have a strong influence on the bioavailability of (poly)phenols.

In terms of (poly)phenol metabolites, the most significant shift due to the beverage consumption was observed in the DHPAA concentration. For men, a strong interaction was observed with the sweetener: for men whose drinks were sweetened with stevia, the DHPAA concentration increased, but for other sweeteners, it decreased. Moreover, regardless of the sweetener, the DPHAA concentration increased in women (with a higher effect for products containing stevia), and the aggregation of DHPAA and its derivatives (Total DHPAA) increased in women with sucralose in their drink. These results suggest a differential metabolism of DHPAA by sex, including a positive support of the effects of alternative sweeteners to sucrose.

The clustering analysis for the (poly)phenol metabolites at the beginning of this study describes the baseline of the participants, which can be considered as representative of the general population. The analysis built two main clusters in terms of population (clusters 3 and 4), leaving the rest with few individuals (clusters 1, 2, 5, and 6). Those clusters corresponded to individuals with low (cluster 3) and high (cluster 4) bioavailability for (poly)phenol metabolites. In cluster 3, the concentrations for every metabolite were below the means of the rest of the clusters, except for DHPAA, which could be related to the presence of women in the cluster. On the other hand, cluster 4 showed a high level of Total CA, which is a common degradation product from anthocyanin due to mammal enzymes [23]. This cluster also showed low levels of TFA-G, DHPAA-G, and Total VA, all of them derivatives of CA, which may indicate that the metabolism of CA is lower for this cluster, indicating in turn, since this cluster was balanced in sexes, a group of volunteers with a high bioavailability for (poly)phenol metabolites.

Looking at the clustering results for (poly)phenol metabolite levels at the end of the study, the six clusters were relevant in terms of the number of individuals. Overall, there were clusters with more women present whose (poly)phenol metabolite bioavailability was enhanced by alternative sweeteners to sucrose: those clusters displayed high levels of Total CA, and, contrary to the second cluster results at the initial time, high levels of CA metabolites (DHPAA and TFA). In addition, regardless of sex, sucrose-absent clusters had higher levels of VA and its derivatives. This supports the hypothesis of an improved bioavailability due to the presence of alternative sweeteners and provides insights into a more active (poly)phenols metabolism after drink consumption in women. In addition, clusters which contained a greater number of men displayed a similar pattern to the second cluster at the beginning of the study (high level of Total CA and low levels of its metabolites).

The analysis of the flavanone metabolites set revealed that the concentration of several metabolites was positively influenced by consuming the drink, according to the ANOVA and *t*-tests. Stevia-sweetened beverages were the most effective in enhancing the bioavailability of ES, HE-G, and Total HE in women, and the bioavailability of NG and Total N in men. It could be discussed whether the observed influences over the metabolite concentrations were due to the sex-isolated factor without relation with the beverages (in the ES case) or due to the drinks regardless of the other factors (in the HE-G, Total HE, NG, and Total N cases) since the ANOVA and *t*-test results were ambiguous. For the ES concentration, the ANOVA was not significant for the drink itself, but it was significant for the sex and sweetener. Moreover, the pairwise *t*-test values were slightly significant. This situation may have occurred due to the weak significance level of the ANOVA (1.20 × 10^−2^ for sex, and 2.80 × 10^−2^ for sweetener) or the high number of levels combined in factors, causing penalizations in the *p*-value by the Benjamini–Hochberg correction [24]. Regarding the HE-G, Total HE, NG, and Total N cases, the ANOVA was not significant for the sex or sweetener, but the *t*-test stated a strong influence of determined levels (and combinations of levels) of those factors. The reason behind this may rely on the nature of the post-hoc tests in general, which can be powerful enough to determine differences between levels even if the ANOVA is not significant. Additionally, the *p*-values for the sex and sweetener factors in the metabolites listed before were closer to the significant level in the ANOVA than other metabolites’ *p*-values. 

In order to gain a deeper knowledge of the flavanone metabolites’ bioavailability, a clustering analysis was conducted. For the data obtained at the beginning of the trial, the algorithm built two principal clusters (3 and 5) regarding the number of individuals included, both containing more men than women and a less populated cluster with most of the women (cluster 1). This last cluster presented the highest concentrations of N and its derivatives and the lowest in E, HE, and their derivatives. Meanwhile, the principal clusters containing mostly men had high values for the concentration of E, HE, and their derivatives. The results indicated better bioavailability for N in women and greater bioavailability for E and HE in men during the baseline condition before commencing the trial.

For the flavanone metabolites data obtained at the end of the trial, the clustering analysis described six clusters with relevance in the number of individuals and different compositions in the sex and sweetener. In general, sucrose seemed to enhance the bioavailability of all metabolites, this effect being more acute in women, where the clusters with fewer sucrose individuals (clusters 1 and 3) presented an enormous difference compared to the clusters containing more women whose drinks were sweetened with sucrose (cluster 6). Furthermore, in the cluster with a higher percentage of men who consumed alternative sweeteners to sucrose (cluster 3), the concentrations of N and its derivatives were much lower than those obtained for other clusters (albeit still greater than in the baseline). Compared to the pre-intake clusters, the distribution of the metabolites’ concentrations, in terms of the sex of the volunteers, remained similar. This could lead to the idea that the sex of the consumer and/or the sweetener added to the beverages are unrelated to the bioavailability of flavanone metabolites. However, three major points led to the rejection of this idea. Discrepancies in metabolite concentrations were observed between the clusters containing mainly the same sex but different sweeteners, which demonstrated effects due to the consumption of the drinks. Additionally, the values of E and EG in the cluster containing women who ingested sucrose-sweetened beverages were lower than the values in the cluster whose composition was balanced in the sweeteners, representing a shift from the initial situation. Finally, according to the results from the ANOVA, it was beyond any doubt that there was an improvement in the bioavailability of flavanone metabolites related to the sex of the consumer (as seen in ES with women) and related to the sweetener (such as stevia and ES or E and sucrose). Moreover, past work, by Agulló et al. and Hernández-Prieto et al., demonstrated the influence of the sweetener and sex on bioavailability [8,9].

By putting together and analyzing the results of previous studies [8,9,25], a major contrast could be detected: the most significant metabolites obtained in the urine samples were for the most part derived from flavanones, while, for plasma, they were predominantly derived from other (poly)phenols (as anthocyanins). Regarding specific variations and similitudes in metabolite concentrations, NG was largely more favored in the plasma samples from women, while the positive influence in the urine samples was spotted in samples from men, both related to stevia consumption. On the other hand, DHPAA in both cases is relevant for men with stevia-sweetened drinks.

It is worth noting that sucralose was almost absent in the significance test from the urine samples, indicating that sucralose almost had no impact on the concentration of metabolites found in the urine. It is also noteworthy that the main analyses present in this work (ANOVA and clustering) did not agree on which variables (understood as metabolites) were the most relevant for their procedures.

As mentioned before, it is not very common to use sex as a factor to describe metabolism, but the present work aims to take that approach. Finally, the study of the interaction between the sex and sweetener can provide even more insight into the differential processing in the catabolism of flavanones and other (poly)phenols (as anthocyanins), which would enable us to better understand how the bioavailability, bioaccessibility, and bioactivity of the metabolites of those compounds work.

## 5. Conclusions

The bioavailability of (poly)phenols depending on the sex of the consumer has been measured in urine samples from a longitudinal intervention trial consisting of daily intake, for 60 days, of a (poly)phenol-rich beverage based on citrus fruits (rich in flavanones) and maqui-berry (rich in anthocyanins), sweetened with sucrose, sucralose, or stevia.

The results indicate that the sex, sweetener, and their interaction affect the bioavailability of phenolic compounds in the beverages. These results agree with previous works, which used a different approach based on plasma samples. Several metabolites were listed to have an influence on their concentration present in urine related to the sex and sweetener: ES, HE-G, and Total HE when related to stevia and women, NG and Total N when paired with stevia and men for flavanone metabolites, and DHPAA when related to stevia and men for (poly)phenol metabolites. These results underline stevia as a promising alternative to sucrose.

The results of this paper pave the way for future research aiming to search for patterns in metabolic behavior shifts via maqui–citrus beverage consumption. Some metabolic pathways related to (poly)phenols in urine have already been identified in this study, and how they are affected by the intervention was described. Namely, the clustering analysis regarding the concentrations of (poly)phenol metabolites at the initial time divided the participants in two main groups with different bioavailability of CA with respect to their metabolites (TFA, DHPAA, and VA). Regarding the end of the trial, it was discovered that the group containing more CA and less TFA, DHPAA, and VA contained the majority of men, and vice versa for women. This pattern may suggest a sex-dependent regulation in the metabolic pathways for polyphenolic compounds.

## Figures and Tables

**Table 3:** Metabolites selected by Boruta algorithm. Abbreviations: Eriodictyol (E), Homoeriodyctiol (HE), total amount of HE and its derivatives (Total HE), Naringenin-Gucuronide (N-G), Naringenin-Di-Glucuronide (N-GG), total amount of Naringenin (Total N).

Sample Time	(Poly)phenol Metabolites Selected
Initial	CA-G, CA-GS, DHPAA-SS, VA, Total VA
Final	CA, Total CA, DHPAA, TFA-G
	**Flavanone metabolites selected**
Initial	E, Total HE, N-GG, Total N
Final	E, HE, N-G, N-GG, Total N

## Data Availability

No new data were created or analyzed in this study. Data sharing is not applicable to this article.

## Supplementary Materials

The following supporting information can be downloaded at: https://www.mdpi.com/article/10.3390/metabo13050653/s1, Figure S1. Zoom on CA-GS cluster analyses results; Table S1. Descriptive statistics; Table S2. Pairwise *t*-test results from (poly)phenols metabolites; Table S3. Pairwise *t*-test results from flavanones; Table S4. Summary of cluster features; Table S5. (Poly)phenols metabolite concentration values and more information about volunteer 66, who was assigned a cluster by the clustering with no one else.
