# Supplementary material for: Differences Due to Sex and Sweetener on the Bioavailability of (Poly)phenols in Urine Samples: A Machine Learning Approach
Source: Metabolites. 2023 May 11;13(5):653. doi: 10.3390/metabo13050653 (PMC10221303; doi:10.3390/metabo13050653)
Supplement: Supplementary file 1 [file metabolites-13-00653-s001.zip › metabolites-2366250-supplementary.pdf]

Supplementary material for "Differences due to sex and sweetener on the bioavailability of (poly)phenols in urine samples: a machine learning approach"

Diego Hernández-Prieto [1], Alberto Garre [2,3], Vicente Agulló [4], Cristina García-Viguera [1,3,*] and Jose A. Egea[5]

[1] Lab Fitoquimica y Alimentos Saludables (LabFAS), Department of Food Science and Technology (CEBAS-CSIC), Campus Universitario Espinardo 25, 30100 Murcia, Spain
[2] Agronomic Engineering Department, Universidad Politécnica de Cartagena (UPCT), Paseo Alfonso XIII, 48, 30203 Cartagena, Spain
[3] Associated Unit of R&D and Innovation CEBAS-CSIC+UPCT on "Quality and Risk Assessment of Foods", CEBAS-CSIC, Campus Universitario de Espinardo 25, 30100 Murcia, Spain
[4] Human Nutrition Unit, Department of Food & Drug, University of Parma, 43125 Parma, Italy.
[5] Group of Fruit Breeding, Department of Plant Breeding, CEBAS-CSIC, Campus Universitario de Espinardo 25, 30100 Murcia, Spain
* Correspondence: cgviguera@cebas.csic.es

Table of contents

Table S1. List of flavonoids present in the beverages 2
Anthocyanins in decreasing concentration order 2
Flavanones in decreasing concentration order 2
Table S2. Descriptive statistics 3
Metabolites derived exclusively from flavanones 3
Metabolites derived from (poly)phenols 4
Table S3. Pairwise T-test results from (poly)phenol metabolites 5
Table S4. Pairwise T-test results from flavanone metabolites 7
Table S5. Distribution of values of metabolites concentrations from different families at different sampling times: 10
Table S6. (Poly)phenol metabolites concentration values and more information about the volunteer 66, who was assigned a cluster by the clustering with no one else. 20
Figure S1. Zoom on CA-GS clusters analyses results 21

Table S1. List of flavonoids present in the beverages

Anthocyanins in decreasing concentration order

Glucoside (glc), sambubioside (sam), rutinoside (rut),

Compound	Concentration (mg/100 mL)
Delphinidin 3,5-*O*-di-glc	5.09
Delphinidin 3-*O*-sam-5-O-glc	4.48
Delphinidin 3-*O*-glc	4.48
Cyanidin 3-*O*-sam-5-*O*-glc	2.09
Cyanidin 3,5-*O*-di-glc	2.09
Delphinidin 3-*O*-sam	1.51
Cyanidin 3-*O*-glc	0.81
Cyanidin 3-*O*-sam	0.51

Flavanones in decreasing concentration order

Compound	Concentration (mg/100 mL)
Hesperidin (hesperetin 7-*O*-rut)	9.11
Eriocitrin (eriodictyol 7-*O*-rut)	1.91
Narirutin (naringenin 7-*O*-rut)	1.73
O-tri-glycosyl-naringenin	0.19

Table S2. Descriptive statistics

Metabolites derived exclusively from flavanones

Eriodictyol (E), E-Glucuronide (EG), E-Sulfate (ES), total amount of E and its derivatives (Total E), Homoe-riodyctiol (HE), HE-Glucuronide (HE-G), HE-Di-Glucuronide (HE-GG), total amount of HE and its derivatives (Total HE), Naringenin-Glucuronide (NG), Naringenin-Sulfate (NS), total amount of Naringenin (Total N)

	**Minimum**	**Q1**	**Mean**	**Trimmed Mean**	**Median**	**Mode**	**Variance**	**Desviation**	**Q3**	**Maximum**	**Symmetry**	**Kurtosis**
**E**	0	0.04056325	0.08935155	0.08086382	0.06506279	0.07379087	0.00915952	0.09570537	0.10483815	1	5.20049085	40.1483766
**EG.1**	0	0.00553683	0.03573123	0.02769077	0.01568355	0.00625348	0.00590074	0.07681626	0.04030376	1	8.58795341	98.449276
**E.S**	0	0.06106193	0.15026867	0.13552852	0.10423892	0.10512323	0.02511006	0.15846155	0.18598444	1	2.8836635	10.2322217
**Total.E**	0	0.00565243	0.03616739	0.02813268	0.01609088	0	0.00590199	0.0768244	0.04080619	1	8.57549361	98.233469
**HE**	0	0.56168122	0.59967686	0.60506954	0.62117904	0.66593886	0.01448162	0.12033959	0.66593886	1	-1.60615983	6.09156837
**HE.G**	0	0.00179312	0.0383656	0.02871144	0.00622817	0.008494	0.00871553	0.093357	0.02364356	1	5.61900805	45.9934719
**HE.GG**	0	0.71535326	0.75508696	0.76466021	0.78396739	0.82065217	0.01949486	0.13962399	0.83627717	1	-2.31362027	7.4725663
**Total.HE**	0	0.00318012	0.03985776	0.03021819	0.00736134	0.00266597	0.00871123	0.09333395	0.02567493	1	5.60248573	45.760395
**N**	0	0.01367548	0.04460892	0.03819554	0.02690877	0.02904509	0.00527926	0.07265851	0.04722334	1	9.39657463	117.233473
**N.G**	0	0.01813239	0.06738374	0.05931254	0.03802517	0	0.00839378	0.09161757	0.08696305	1	5.09535352	42.5573071
**N.GG**	0	0.00790832	0.03486689	0.02775062	0.01680831	0.01680831	0.0053774	0.07333075	0.03793561	1	9.61015401	118.511243
**N.S**	0	0.03027544	0.09592629	0.08254661	0.05764904	0.05147347	0.01634186	0.12783527	0.11213664	1	3.94937443	20.4656351
**Total.N**	0	0.0218553	0.07180752	0.06404426	0.04290314	0	0.00834264	0.09133803	0.09248485	1	5.00971909	41.9939841

Metabolites derived from (poly)phenols

Caffeic Acid (CA), CA-G Glucuronide (CA-G), CA-Glucuronide-Sulfate (CA-GS), total amount of CA and its derivatives (Total CA), 3,4-Dihydroxyphenylacetic acid (DHPAA), DHPAA-Glucuronide (DHPAA-G), DHPAA-Glucuronide-Sulfate (DHPAA-GS), DHPAA-Di-Sulfate (DHPAA-SS), total amount of DHPAA and its derivatives (Total DHPAA), Trans Ferulic Acid- Glucuronide (TFA-G), Trans Ferulic Acid-Suldate (TFA-S), total amount of TFA and its derivatives (Total TFA), Vanillic Acid (VA), VA-Di-Glucuronide (VA-GG) and VA-Di-Sulfate (VA-SS), VA-Glucuronide-Sulfate (VA-GS), total amount of VA and its derivatives (Total VA).

	**Minimum**	**Q1**	**Mean**	**Trimmed Mean**	**Median**	**Mode**	**Variance**	**Desviation**	**Q3**	**Maximum**	**Symmetry**	**Kurtosis**
**CA**	0	0.0159292	0.04314784	0.03575245	0.02654867	0.02558563	0.00562074	0.07497159	0.04385251	1	8.92511915	104.959374
**CA-G**	0	0.01572282	0.07058736	0.05553775	0.03414912	0.00369949	0.01487555	0.12196535	0.08693796	1	5.03328626	29.5817371
**CA-GS**	0	0.0587406	0.14275959	0.13345687	0.11265588	0.06817525	0.01551167	0.12454587	0.18150846	1	2.53668153	10.4350493
**Total CA**	0	0.02178237	0.07106744	0.06271368	0.0426157	0.00449818	0.00883698	0.0940052	0.08375585	1	4.8269179	38.0213099
**DHPAA**	0	0.05781866	0.16246957	0.15257918	0.12220762	0.12746386	0.02121625	0.145658	0.2217477	1	1.92787548	5.6724837
**DHPAA-G**	0	0.02301587	0.05669927	0.05053278	0.04345238	0.0844367	0.00541502	0.07358679	0.06944444	1	8.72562466	105.818761
**DHPAA-GS**	0	0.03375734	0.08436188	0.07595087	0.05418941	0.03375734	0.00934302	0.09665929	0.09676589	1	4.44124344	32.4041848
**DHPAA-SS**	0	0.00479124	0.02136391	0.01605516	0.00924025	0.00684463	0.00429104	0.06550607	0.01950719	1	13.3980016	196.345703
**Total DHPAA**	0	0.04356653	0.14002936	0.12489691	0.09240631	0.0870681	0.02587688	0.16086292	0.176226	1	2.75946122	9.47962219
**TFA-G**	0	0.04440976	0.14111662	0.12600996	0.09314717	0.00075734	0.02586815	0.16083578	0.1769872	1	2.75679551	9.46443216
**TFA-S**	0	0.01243105	0.0525921	0.04133032	0.02484545	0.0213858	0.00929922	0.09643248	0.05204879	1	5.71010241	43.3276199
**Total TFA**	0	0.06714109	0.18145231	0.17047678	0.14356436	0.09742955	0.02543628	0.15948756	0.24752475	1	1.86667801	4.75314642
**VA**	0	0.08139339	0.16916142	0.15913882	0.13259109	0.09483957	0.0192093	0.13859761	0.20175439	1	2.18467029	7.05770251
**VA-GS**	0	0.02136991	0.0577446	0.05113761	0.04217992	0.03538273	0.00585312	0.07650571	0.07269504	1	7.9085561	90.4193506
**Total VA**	0	0.02199914	0.06487771	0.05375034	0.03768678	0.02064076	0.00935345	0.09671321	0.07080559	1	5.56026622	41.2886263

Table S3. Pairwise T-test results from (poly)phenol metabolites

Male (M), female (F), Caffeic Acid (CA), CA-G Glucuronide (CA-G), CA-Glucuronide-Sulfate (CA-GS), total amount of CA and its derivatives (Total CA), 3,4-Dihydroxyphenylacetic acid (DHPAA), DHPAA-Glucuronide (DHPAA-G), DHPAA-Glucuronide-Sulfate (DHPAA-GS), DHPAA-Di-Sulfate (DHPAA-SS), total amount of DHPAA and its derivatives (Total DHPAA), Trans Ferulic Acid-Glucuronide (TFA-G), Trans Ferulic Acid-Suldate (TFA-S), total amount of TFA and its derivatives (Total TFA), Vanillic Acid (VA), VA-Di-Glucuronide (VA-GG) and VA-Di-Sulfate (VA-SS), VA-Glucuronide-Sulfate (VA-GS), total amount of VA and its derivatives (Total VA).

		Sweetener			
	**caffeic acid**	SA	ST	SU	1-way sex
Sex	M				
	F	0.03			0.015
	1-way sweetener				

		Sweetener			
	**CA-Gluc**	SA	ST	SU	1-way sex
Sex	M		0.003		
	F		0.025		0.005
	1-way sweetener		0.002		0.003

		Sweetener			
	**DHPAA-Gluc**	SA	ST	SU	1-way sex
Sex	M				
	F				
	1-way sweetener				

		Sweetener			
	**DHPAA-di-Gluc**	SA	ST	SU	1-way sex
Sex	M				
	F				
	1-way sweetener				

		Sweetener			
	**DHPAA-G-Sulfate**	SA	ST	SU	1-way sex
Sex	M				
	F				
	1-way sweetener				

		Sweetener			
	**DPHAA-di-Sulfate**	SA	ST	SU	1-way sex
Sex	M				0.049
	F				
	1-way sweetener				

		Sweetener			
	**TOTAL DHPAA**	SA	ST	SU	1-way sex
Sex	M				
	F			0.02	0.043
	1-way sweetener				

Table S4. Pairwise T-test results from flavanone metabolites

Male (M), Female (H), Sucrose (SA), Sucralose (SU), Stevia (ST), Eriodictyol (E), E-Glucuronide (EG), E-Sulfate (ES), total amount of E and its derivatives (Total E), Homoe-riodyctiol (HE), HE-Glucuronide (HE-G), HE-Di-Glucuronide (HE-GG), total amount of HE and its derivatives (Total HE), Naringenin-Glucuronide (NG), Naringenin-Sulfate (NS), total amount of Naringenin (Total N)

		Sweetener			
	**Eriodyctiol**	SA	ST	SU	1-way sex
Sex	M				
	F	0.032			
	1-way sweetener	0.008			

		Sweetener			
	**EG**	SA	ST	SU	1-way sex
Sex	M	0.044			
	F				
	1-way sweetener				

		Sweetener			
	**ES**	SA	ST	SU	1-way sex
Sex	M				
	F	0.034	0.015		0.000632
	1-way sweetener		0.007		

		Sweetener			
	**TOTAL E**	SA	ST	SU	1-way sex
Sex	M	0.043			
	F				
	1-way sweetener				

		Sweetener			
	**HE**	SA	ST	SU	1-way sex
Sex	M	0.018		0.015	0.005
	F				
	1-way sweetener			0.042	0.007

		Sweetener			

	HE-G	SA	ST	SU	1-way sex
Sex	M	0.016	0.016		0.000461
	F		0.003		
	1-way sweetener	0.015	0.000235		0.001

		Sweetener			
	HE-GG	SA	ST	SU	1-way sex
Sex	M				0.041
	F				
	1-way sweetener				0.008

		Sweetener			
	TOTAL HE	SA	ST	SU	1-way sex
Sex	M	0.017	0.016	0.026	0.000311
	F		0.002		0.05
	1-way sweetener	0.015	0.000142		0.000602

		Sweetener			
	Naringenine	SA	ST	SU	1-way sex
Sex	M				
	F				
	1-way sweetener				

		Sweetener			
	NG	SA	ST	SU	1-way sex
Sex	M	0.032	0.000548		0.017
	F	0.015	0.013		0.03
	1-way sweetener	0.002	0.0000375		0.003

		Sweetener			
	NGG	SA	ST	SU	1-way sex
Sex	M				
	F				
	1-way sweetener				

		Sweetener			
	NS	SA	ST	SU	1-way sex
Sex	M	0.021		0.048	
	F				
	1-way sweetener		0.049		

		Sweetener			
	**TOTAL N**	SA	ST	SU	1-way sex
Sex	M		0.0004		0.033
	F	0.014	0.027		0.036
	1-way sweetener	0.003	0.0000682		0.004

Table S5. Distribution of values of metabolites concentrations from different families at different sampling times:

Caffeic Acid (CA), CA-G Glucuronide (CA-G), CA-Glucuronide-Sulfate (CA-GS), total amount of CA and its derivatives (Total CA), 3,4-Dihydroxyphenylacetic acid (DHPAA), DHPAA-Glucuronide (DHPAA-G), DHPAA-Glucuronide-Sulfate (DHPAA-GS), DHPAA-Di-Sulfate (DHPAA-SS), total amount of DHPAA and its derivatives (Total DHPAA), Trans Ferulic Acid- Glucuronide (TFA-G), Trans Ferulic Acid-Suldate (TFA-S), total amount of TFA and its derivatives (Total TFA), Vanillic Acid (VA), VA-Di-Glucuronide (VA-GG) and VA-Di-Sulfate (VA-SS), VA-Glucuronide-Sulfate (VA-GS), total amount of VA and its derivatives (Total VA). Eriodictyol (E), E-Glucuronide (EG), E-Sulfate (ES), total amount of E and its derivatives (Total E), Homoe-riodyctiol (HE), HE-Glucuronide (HE-G), HE-Di-Glucuronide (HE-GG), total amount of HE and its derivatives (Total HE), Naringenin-Glucuronide (NG), Naringenin-Sulfate (NS), total amount of Naringenin (Total N)

**Metabolites from (poly)phenols at initial time (day 0)**							
**cluster**	**metabolite**	**min**	**q1**	**median**	**mean**	**q3**	**max**
1	CA	0.01061947	0.0159292	0.02123894	0.02729605	0.02558563	0.11504425
1	CA.G	0.06289129	0.0971827	0.098321	0.11011058	0.11895276	0.19578828
1	CA.GS	0.002	0.002	0.003	0.00392857	0.00475	0.009
1	Total.CA	0.359	0.39525	0.574	0.61192857	0.745	1.153
1	DHPAA	0.01268797	0.06578947	0.11083922	0.11740507	0.16952538	0.22368421
1	DHPAA.G	0.01019241	0.03292382	0.04938898	0.05371344	0.06292555	0.16209048
1	DHPAA.GS	0.02102497	0.05519054	0.13666229	0.18002628	0.29500657	0.42838371
1	DHPAA.SS	0.00714286	0.02787698	0.03869048	0.06380386	0.06210318	0.20436508
1	Total.DHPAA	0.0186425	0.04488104	0.06495262	0.06866389	0.08141823	0.17813369
1	TFA.G	0.00239562	0.00504791	0.01163587	0.09137577	0.04218001	1
1	TFA.S	0	0.03881303	0.05454073	0.08584453	0.12875056	0.21764317
1	Total.TFA	0.00075734	0.03985221	0.0552965	0.08932339	0.13042714	0.21848121
1	VA	0.00241622	0.01045642	0.02003799	0.02969932	0.03736815	0.09216644
1	VA.GG	0.00247525	0.03929455	0.10334158	0.16796323	0.20792079	0.86138614
1	VA.GS	0.03070175	0.06123482	0.10307018	0.1996578	0.2462888	1
1	VA.SS	0	0.02967525	0.05655095	0.06224536	0.07316163	0.16759985
1	Total.VA	0.01403108	0.02684044	0.03935162	0.04483839	0.04796988	0.11183448
2	CA	0.0159292	0.02477876	0.04266667	0.22694041	0.05132743	1
2	CA.G	0.50540694	0.80961867	0.81331816	0.79658509	0.85458167	1
2	CA.GS	0.002	0.002	0.002	0.0038	0.005	0.008
2	Total.CA	1.81	2.88	3.018	2.9314	3.418	3.531
2	DHPAA	0.04981203	0.15413534	0.1799812	0.22302632	0.31907895	0.41212406
2	DHPAA.G	0.08283931	0.11479459	0.13065523	0.16525741	0.14708788	0.35091004
2	DHPAA.GS	0.03022339	0.04599212	0.0499343	0.08883049	0.05519054	0.26281209
2	DHPAA.SS	0.00595238	0.01309524	0.07738095	0.07015873	0.1	0.15436508
2	Total.DHPAA	0.09661139	0.14081723	0.15063343	0.18635172	0.15784324	0.38585333
2	TFA.G	0.00308008	0.00787132	0.01060917	0.01444216	0.02258727	0.02806297
2	TFA.S	0.04708023	0.08145918	0.10998019	0.25093933	0.15633493	0.85984213

2	Total.TFA	0.04837226	0.08303671	0.11083102	0.2518452	0.15698536	0.86000065
2	VA	0.07636933	0.09966506	0.10548066	0.10397094	0.10628052	0.13205912
2	VA.GG	0.03960396	0.06806931	0.17326733	0.22376238	0.37004951	0.46782178
2	VA.GS	0.10053981	0.13529015	0.1437247	0.18466787	0.21626181	0.32752287
2	VA.SS	0.01007839	0.02239642	0.02612915	0.03210153	0.02948862	0.07241508
2	Total.VA	0.08978564	0.10890236	0.11551204	0.11424974	0.11879204	0.13825664
3	CA	0.00176991	0.01769912	0.02558563	0.03811782	0.04252507	0.17121565
3	CA.G	0.00142288	0.01017359	0.01906659	0.02268901	0.03009391	0.06829824
3	CA.GS	0.001	0.002	0.002	0.00279167	0.003	0.009
3	Total.CA	0.024	0.089	0.1705	0.25839583	0.25975	1.917
3	DHPAA	0.00234962	0.05321899	0.08952068	0.10789946	0.15695489	0.3087406
3	DHPAA.G	0.00228809	0.0224454	0.04144566	0.04689963	0.05805036	0.14126365
3	DHPAA.GS	0	0.04862024	0.14651774	0.16714623	0.20597898	1
3	DHPAA.SS	0.02777778	0.05426587	0.0640873	0.07882838	0.08545054	0.26746032
3	Total.DHPAA	0.00937275	0.03549542	0.05112525	0.05990996	0.06866052	0.15431558
3	TFA.G	0.00136893	0.00479124	0.00906913	0.01254005	0.01753936	0.04346338
3	TFA.S	0.00123439	0.04170953	0.069099	0.11493152	0.11821219	0.55338755
3	Total.TFA	0.00195826	0.04240823	0.06991853	0.11586777	0.11927804	0.55398198
3	VA	0.00056656	0.00866091	0.02033794	0.02317457	0.03062355	0.06487144
3	VA.GG	0.01113861	0.10232769	0.17079208	0.22891391	0.29641089	1
3	VA.GS	0.04183536	0.10011808	0.17847503	0.2171982	0.31595817	0.75809717
3	VA.SS	0.00335946	0.03331467	0.05617768	0.0707488	0.09257186	0.33370661
3	Total.VA	0.00723917	0.02585479	0.03767021	0.03979441	0.05195391	0.08930524
4	CA	0.00353982	0.01061947	0.01946903	0.02740215	0.02743363	0.17168142
4	CA.G	0.0011383	0.00839499	0.01394422	0.01887467	0.02675014	0.07199772
4	CA.GS	0.001	0.002	0.002	0.00253488	0.003	0.008
4	Total.CA	0.018	0.086	0.168	0.22472093	0.2995	0.959
4	DHPAA	0.00422932	0.06817525	0.10526316	0.10403205	0.12429511	0.33646617
4	DHPAA.G	0.00189808	0.00963339	0.02561102	0.03040731	0.03776651	0.11583463
4	DHPAA.GS	0.00788436	0.06633149	0.10512484	0.14664509	0.18593955	0.64914586
4	DHPAA.SS	0.00396825	0.00833333	0.01984127	0.02040648	0.02797619	0.0468254
4	Total.DHPAA	0.00576784	0.02146205	0.03375734	0.04001262	0.04977341	0.13214543
4	TFA.G	0	0.00410678	0.00821355	0.01491347	0.01591376	0.08829569
4	TFA.S	0.0017	0.01747101	0.06664645	0.1107253	0.1692311	0.57153531
4	Total.TFA	0.00238021	0.01823562	0.06733817	0.11174913	0.16992502	0.57237447
4	VA	0.00098315	0.00649881	0.01308094	0.01566448	0.02028795	0.08321808
4	VA.GG	0	0.05940594	0.09742955	0.1518077	0.18502475	0.52952617
4	VA.GS	0.0111336	0.06747638	0.11707153	0.13367713	0.17004049	0.40789474
4	VA.SS	0	0.02090332	0.03538273	0.04497653	0.0628966	0.21836506
4	Total.VA	0.00536726	0.01456118	0.01986217	0.02472707	0.03014942	0.09687573
5	CA	0.00884956	0.01967074	0.03274336	0.055903	0.05309735	0.17168142
5	CA.G	0.00085373	0.00796813	0.02518497	0.04925197	0.08992601	0.16932271
5	CA.GS	0.001	0.002	0.002	0.00321429	0.00475	0.007
5	Total.CA	0.028	0.11025	0.208	0.23528571	0.36075	0.698
5	DHPAA	0.03007519	0.05944549	0.10361842	0.17852947	0.22450658	0.6306391

5	DHPAA.G	0.03278731	0.09214119	0.13454768	0.17092417	0.23575143	0.39121165
5	DHPAA.GS	0.02233903	0.05617608	0.18856767	0.21834858	0.30124836	0.77923785
5	DHPAA.SS	0.01944444	0.03720238	0.05238095	0.06726757	0.06321429	0.24920635
5	Total.DHPAA	0	0.088127	0.13416675	0.1776886	0.25198913	0.42867443
5	TFA.G	0.00136893	0.00530459	0.00992471	0.02094945	0.02600958	0.091718
5	TFA.S	0.03603565	0.05215315	0.18621485	0.30152319	0.41086104	0.93770641
5	Total.TFA	0.03703383	0.0530326	0.1872302	0.30258515	0.41247877	0.93879627
5	VA	0.0869174	0.11811168	0.14169069	0.15961836	0.16907317	0.33023946
5	VA.GG	0.01237624	0.02011139	0.18069307	0.16354314	0.26732673	0.39480198
5	VA.GS	0.07388664	0.10686572	0.18252362	0.2375972	0.32752287	0.67678812
5	VA.SS	0.00970511	0.03107503	0.06281718	0.06911291	0.08967899	0.21127286
5	Total.VA	0.11431932	0.13164695	0.15215519	0.17238772	0.18400673	0.33772322
6	CA	0.08849558	0.08849558	0.08849558	0.08849558	0.08849558	0.08849558
6	CA.G	0.17842914	0.17842914	0.17842914	0.17842914	0.17842914	0.17842914
6	CA.GS	0.009	0.009	0.009	0.009	0.009	0.009
6	Total.CA	0.678	0.678	0.678	0.678	0.678	0.678
6	DHPAA	0.06907895	0.06907895	0.06907895	0.06907895	0.06907895	0.06907895
6	DHPAA.G	0.15364015	0.15364015	0.15364015	0.15364015	0.15364015	0.15364015
6	DHPAA.GS	0.44546649	0.44546649	0.44546649	0.44546649	0.44546649	0.44546649
6	DHPAA.SS	1	1	1	1	1	1
6	Total.DHPAA	0.23084252	0.23084252	0.23084252	0.23084252	0.23084252	0.23084252
6	TFA.G	0.04483231	0.04483231	0.04483231	0.04483231	0.04483231	0.04483231
6	TFA.S	0.32029279	0.32029279	0.32029279	0.32029279	0.32029279	0.32029279
6	Total.TFA	0.32206342	0.32206342	0.32206342	0.32206342	0.32206342	0.32206342
6	VA	0.03522688	0.03522688	0.03522688	0.03522688	0.03522688	0.03522688
6	VA.GG	0.54579208	0.54579208	0.54579208	0.54579208	0.54579208	0.54579208
6	VA.GS	0.70006748	0.70006748	0.70006748	0.70006748	0.70006748	0.70006748
6	VA.SS	1	1	1	1	1	1
6	Total.VA	0.12177385	0.12177385	0.12177385	0.12177385	0.12177385	0.12177385

**Metabolites from (poly)phenols at final time (day 60)**							
**clusters**	**metabolite**	**min**	**q1**	**median**	**mean**	**q3**	**max**
1	CA	0.00353982	0.01106195	0.02606715	0.04502763	0.04469027	0.2460177
1	CA.G	0.0130905	0.04894707	0.07882755	0.08577777	0.11354582	0.18412066
1	CA.GS	0.002	0.003	0.005	0.00523077	0.006	0.015
1	Total.CA	0.505	0.558	0.6015	0.61342308	0.682	0.803
1	DHPAA	0	0.04452538	0.11513158	0.13253687	0.16459117	0.53524436
1	DHPAA.G	0.00449818	0.03855304	0.06842174	0.09071463	0.11284451	0.28780551
1	DHPAA.GS	0.03547963	0.08571346	0.1478318	0.18916339	0.28975033	0.51642576
1	DHPAA.SS	0.0031746	0.02172619	0.03373016	0.04668133	0.06636905	0.13888889
1	Total.DHPAA	0.01104645	0.05086131	0.08000309	0.10381724	0.1234486	0.29127614
1	TFA.G	0.00102669	0.00496236	0.01608487	0.02481177	0.03413758	0.09582478
1	TFA.S	0.01531082	0.04450857	0.09132351	0.15526289	0.16017076	1
1	Total.TFA	0.00113601	0.04604075	0.09269277	0.15543157	0.16109663	1

1	VA	0.00499908	0.02104357	0.02986952	0.07689897	0.09292047	0.47581277
1	VA.GG	0.01237624	0.08469155	0.1595222	0.18999452	0.2237005	0.75371287
1	VA.GS	0.03778678	0.08383941	0.1548583	0.17667505	0.22494939	0.55904184
1	VA.SS	0.00223964	0.02743561	0.03579513	0.04859958	0.07190878	0.14483016
1	Total.VA	0.00757049	0.02664994	0.05010271	0.08855915	0.10465742	0.50031475
2	CA	0.01946903	0.03185841	0.04070797	0.07869299	0.05132743	0.32566372
2	CA.G	0.01109846	0.05549232	0.11582243	0.11227617	0.1445646	0.24957314
2	CA.GS	0.002	0.004	0.006	0.00630769	0.007	0.012
2	Total.CA	0.715	0.801	0.904	0.90776923	0.981	1.123
2	DHPAA	0.05545113	0.17246241	0.31907895	0.32967033	0.39473684	1
2	DHPAA.G	0.00356214	0.04425377	0.11391056	0.11723469	0.19451378	0.30426417
2	DHPAA.GS	0.01182654	0.11316379	0.2325887	0.25558959	0.33508541	0.70302234
2	DHPAA.SS	0.02460318	0.05396825	0.0844367	0.08026743	0.11190476	0.13452381
2	Total.DHPAA	0.00898651	0.06813266	0.13466886	0.14394654	0.21935833	0.36726233
2	TFA.G	0.00308008	0.0065024	0.01471595	0.03051124	0.04175223	0.10061602
2	TFA.S	0.00213312	0.11000184	0.18127186	0.19074721	0.25868136	0.37545072
2	Total.TFA	0.0029969	0.11110149	0.18301615	0.19221157	0.25944238	0.37680815
2	VA	0.00886504	0.03107764	0.05377348	0.0954748	0.0951159	0.55018247
2	VA.GG	0.06435644	0.1595222	0.20668317	0.25953172	0.32425743	0.62376238
2	VA.GS	0.08670715	0.13719709	0.30904184	0.27837393	0.38090418	0.451417
2	VA.SS	0.00037327	0.04031355	0.06308324	0.07313291	0.09854423	0.21724524
2	Total.VA	0.03188881	0.0421098	0.08269556	0.11446586	0.11711891	0.57734486
3	CA	0.01769912	0.04469027	0.0539823	0.0619469	0.06769912	0.14690266
3	CA.G	0.07968128	0.14591634	0.17401821	0.22748293	0.28564314	0.43881616
3	CA.GS	0.002	0.0045	0.0065	0.00625	0.00725	0.012
3	Total.CA	1.288	1.31525	1.6385	1.715125	2.0195	2.347
3	DHPAA	0.03242481	0.17340226	0.22485902	0.25881109	0.28418703	0.61889098
3	DHPAA.G	0.02332527	0.03114925	0.06540562	0.07676186	0.10462819	0.1599064
3	DHPAA.GS	0.06742773	0.10640991	0.30486202	0.29471489	0.4609067	0.53876478
3	DHPAA.SS	0.01825397	0.0468254	0.06531746	0.07972222	0.09365079	0.20634921
3	Total.DHPAA	0.01599032	0.04705685	0.08949171	0.09808232	0.13358739	0.19870739
3	TFA.G	0.00376455	0.02027721	0.04004107	0.04538843	0.05039357	0.13826147
3	TFA.S	0.02242483	0.10344277	0.16900371	0.21069294	0.26987212	0.57898498
3	Total.TFA	0.023207	0.10773405	0.17044434	0.21265782	0.271622	0.58049963
3	VA	0.00761527	0.01048141	0.03291063	0.04281715	0.0494576	0.14555665
3	VA.GG	0.01113861	0.06497525	0.13242574	0.1634901	0.28929455	0.33267327
3	VA.GS	0.11032389	0.17341431	0.20630904	0.245361	0.2817139	0.51551957
3	VA.SS	0.02836879	0.056271	0.075028	0.08328667	0.11440836	0.13736469
3	Total.VA	0.02244641	0.03839082	0.04481828	0.06063844	0.0637155	0.16891959
4	CA	0.00530974	0.02876106	0.04159292	0.05205297	0.06061947	0.26548673
4	CA.G	0.02902675	0.03756403	0.06061468	0.05939877	0.07100171	0.10244735
4	CA.GS	0.002	0.003	0.003	0.00472727	0.005	0.013
4	Total.CA	0.143	0.22425	0.2585	0.2635	0.30075	0.392

4	DHPAA	0.11432427	0.19407895	0.22227444	0.25088891	0.29323308	0.59962406
4	DHPAA.G	0.0099844	0.02518201	0.04222569	0.07036118	0.08195528	0.32943318
4	DHPAA.GS	0.01445467	0.0413929	0.09067017	0.11830277	0.15045992	0.46780552
4	DHPAA.SS	0.00674603	0.0139881	0.03353175	0.04525217	0.06607143	0.1515873
4	Total.DHPA A	0.0295602	0.04345195	0.06183696	0.08844652	0.10733598	0.35392419
4	TFA.G	0.00034223	0.00727242	0.01454483	0.01695601	0.02036277	0.0687885
4	TFA.S	0.00831592	0.05552879	0.11473369	0.12657658	0.17588221	0.31867942
4	Total.TFA	0.00909888	0.05661373	0.11580781	0.12767392	0.17696286	0.31929373
4	VA	0.00779857	0.01448068	0.02312909	0.03324921	0.03564347	0.12739331
4	VA.GG	0.01361386	0.07394802	0.10705446	0.13890581	0.21967822	0.33267327
4	VA.GS	0.03407557	0.05727058	0.09564777	0.09905226	0.12457827	0.22705803
4	VA.SS	0.00447928	0.01465099	0.03691495	0.0424335	0.05963046	0.11048899
4	Total.VA	0.010983	0.02179621	0.03267568	0.04159024	0.04608555	0.13174635
5	CA	0	0.0159292	0.02300885	0.02505071	0.03185841	0.06371681
5	CA.G	0.00369949	0.01166762	0.01792829	0.0201362	0.02390438	0.04610131
5	CA.GS	0	0.002	0.003	0.00351724	0.005	0.011
5	Total.CA	0.028	0.074	0.095	0.10744828	0.155	0.199
5	DHPAA	0.01221805	0.04370301	0.11701128	0.1124801	0.13768797	0.29746241
5	DHPAA.G	0.00447218	0.01406656	0.02659906	0.03486112	0.0473739	0.14968799
5	DHPAA.GS	0.00131406	0.05256242	0.09843859	0.10943771	0.14980289	0.32720105
5	DHPAA.SS	0.00634921	0.0218254	0.03358054	0.03956335	0.05390212	0.10476191
5	Total.DHPA A	0.00592234	0.02168092	0.03185189	0.04457293	0.0683644	0.16368833
5	TFA.G	0.00136893	0.00342231	0.0065024	0.01026694	0.00924025	0.07871321
5	TFA.S	0.00309681	0.0290624	0.08371141	0.09734874	0.1281496	0.31338451
5	Total.TFA	0.00401389	0.02970929	0.08460548	0.09823496	0.12920187	0.31394909
5	VA	0.00354935	0.01338088	0.0260119	0.03016171	0.04120911	0.08828382
5	VA.GG	0.0049505	0.06064356	0.1299505	0.159664	0.18069307	0.61386139
5	VA.GS	0	0.05533063	0.09483957	0.11633981	0.16329285	0.46255061
5	VA.SS	0	0.01717059	0.02500933	0.03402138	0.03844718	0.0862262
5	Total.VA	0.00685817	0.01933207	0.03866415	0.03925251	0.05017725	0.09531856
6	CA	0.00176991	0.01061947	0.02831858	0.03074402	0.04690266	0.07433628
6	CA.G	0	0.04681275	0.0734206	0.07234554	0.10515083	0.12464428
6	CA.GS	0.001	0.002	0.003	0.00381482	0.0045	0.013
6	Total.CA	0.227	0.295	0.359	0.35918519	0.419	0.467
6	DHPAA	0.00704887	0.04182331	0.06672932	0.07875592	0.1050282	0.1912594
6	DHPAA.G	0	0.02085283	0.04383775	0.11213575	0.1025351	1
6	DHPAA.GS	0	0.06767411	0.12483574	0.12765854	0.18134034	0.31406045
6	DHPAA.SS	0	0.02123016	0.04087302	0.04777518	0.06706349	0.13412698
6	Total.DHPA A	0.00733855	0.03165877	0.05798743	0.1218171	0.111237	1
6	TFA.G	0.00136893	0.00633128	0.01060917	0.01231657	0.01694045	0.03353867
6	TFA.S	0.00319427	0.05756716	0.10177255	0.14523742	0.15774799	0.90418286
6	Total.TFA	0.00429519	0.05848273	0.10252194	0.14612618	0.1585325	0.90459704

6	VA	0	0.01308094	0.02902801	0.10989961	0.09773208	1
6	VA.GG	0.02227723	0.06373762	0.14727723	0.15681151	0.24814356	0.33415842
6	VA.GS	0.00910931	0.06039136	0.10188934	0.09790532	0.13124157	0.21738642
6	VA.SS	0.00149309	0.0177305	0.03844718	0.04836761	0.06942889	0.13960433
6	Total.VA	0.01275553	0.02170096	0.03324719	0.11788277	0.10685651	1

Metabolites from flavanones at final time (day 0)							
clusters	metabolite	min	q1	median	mean	q3	max
1	E	0.03185331	0.06410784	0.07379087	0.07164977	0.07968903	0.10493892
1	EG.1	0.00023815	0.00665197	0.01163218	0.03426453	0.05617246	0.12507592
1	E.S	0.00949046	0.03659694	0.07076207	0.0962471	0.14101243	0.29875401
1	Total.E	0.00049985	0.00693481	0.01213505	0.03464735	0.05650525	0.12557095
1	HE	0.40829694	0.51746725	0.56768559	0.56404658	0.62445415	0.72707424
1	HE.G	7.64E-05	0.00144651	0.00415939	0.01370098	0.00957451	0.07832969
1	HE.GG	0.51902174	0.63451087	0.7201087	0.69927536	0.76630435	0.84782609
1	Total.HE	0.00143636	0.00221439	0.00368883	0.01379703	0.00905886	0.08040349
1	N	0.00660918	0.02439383	0.04443571	0.06028579	0.07156787	0.20975789
1	N.G	0.00161611	0.01737543	0.02964691	0.03041721	0.04383871	0.06485884
1	N.GG	0.02700395	0.04695686	0.06350321	0.0623529	0.08036262	0.08815266
1	N.S	0.00525714	0.03956316	0.05147348	0.07995915	0.11969882	0.20628392
1	Total.N	0.0018915	0.02416857	0.03329587	0.03629987	0.04754089	0.07820571
2	E	0.07933638	0.1035268	0.11027731	0.11692862	0.12015144	0.17527815
2	EG.1	0.00237032	0.01077282	0.02438176	0.0334617	0.03007665	0.1118158
2	E.S	0.0214686	0.05402433	0.09728233	0.10753469	0.14987395	0.23046743
2	Total.E	0.00265949	0.01133677	0.02475549	0.03384174	0.03056836	0.11196015
2	HE	0.42358079	0.45796943	0.51855895	0.51965066	0.5720524	0.62227074
2	HE.G	0.00034935	0.00146561	0.00314956	0.0048589	0.00493996	0.02264192
2	HE.GG	0.50815217	0.56182065	0.64266304	0.64285714	0.71059783	0.78804348
2	Total.HE	0.00147989	0.00293529	0.00417306	0.00596618	0.0055931	0.02428753
2	N	0.00877355	0.01743958	0.02905271	0.0319244	0.03893384	0.10481191
2	N.G	0.00740891	0.01360318	0.01768627	0.03084686	0.02652474	0.15677259
2	N.GG	0.00670229	0.03178117	0.04173065	0.0404933	0.04458107	0.08603723
2	N.S	0.00953427	0.02972209	0.08012873	0.08142101	0.10383767	0.29544024
2	Total.N	0.0076276	0.01963545	0.02138641	0.03559069	0.0381229	0.15984223
3	E	0.00994174	0.02842812	0.0405096	0.04154344	0.05428927	0.07379087
3	EG.1	0.00020498	0.00511673	0.01447546	0.03468464	0.04573258	0.30044785
3	E.S	0	0.02784493	0.06383178	0.11636469	0.12112599	0.82772008
3	Total.E	0	0.00429155	0.01477983	0.03494273	0.04617992	0.30065525
3	HE	0.54148472	0.61517467	0.64956332	0.64693331	0.68176856	0.72925764
3	HE.G	0	0.00064138	0.00157205	0.00268338	0.0045524	0.0085262
3	HE.GG	0.66847826	0.77377717	0.81521739	0.81101779	0.84918478	0.95923913
3	Total.HE	0.00102286	0.00229328	0.00296521	0.00401305	0.00522857	0.01040273
3	N	0.00782171	0.01136616	0.01463965	0.02614351	0.02904509	0.13660726
3	N.G	0	0.00898335	0.01389817	0.0151426	0.01980461	0.03605911
3	N.GG	0.0015827	0.00558474	0.01090201	0.01370652	0.01680831	0.05423224
3	N.S	0.0086635	0.02632791	0.0314041	0.04463434	0.05580327	0.1819531
3	Total.N	0.00076408	0.00976314	0.01524625	0.01664234	0.02402947	0.03496149
4	E	0.14324762	0.18855046	0.21309713	0.21581892	0.24619507	0.28981953
4	EG.1	0.00117278	0.00433185	0.00850887	0.0341607	0.03426154	0.20720338
4	E.S	0.03484354	0.10124818	0.15270019	0.26909582	0.31948192	1

4	Total.E	0.00031657	0.00252109	0.00942125	0.03436257	0.03523843	0.2075722
4	HE	0	0.11790393	0.2860262	0.31659389	0.44868996	0.77729258
4	HE.G	0.00029476	0.00655022	0.008494	0.03828508	0.04297489	0.17025109
4	HE.GG	0	0.14945652	0.36413044	0.42366601	0.61005435	0.98913044
4	Total.HE	0	0.00524489	0.00905342	0.03925753	0.04555545	0.17173201
4	N	0	0.01712567	0.02513448	0.05206899	0.09159741	0.12588759
4	N.G	0.00282033	0.0191195	0.04081126	0.04542257	0.06715041	0.1135512
4	N.GG	0.00617136	0.02719003	0.09394701	0.10422884	0.14619662	0.32248665
4	N.S	0.03446457	0.09452934	0.12206821	0.19142033	0.29562032	0.45602182
4	Total.N	0.0099236	0.03287057	0.05429472	0.06094037	0.08276455	0.144725
5	E	0.01522922	0.0404096	0.04930326	0.05051901	0.06087799	0.09841567
5	EG.1	0.00031274	0.00647857	0.02065057	0.03652396	0.04364693	0.30851839
5	E.S	0.00362933	0.09263199	0.13216988	0.16373989	0.19075565	0.7891188
5	Total.E	0.0008329	0.00697091	0.0210943	0.0370282	0.04417755	0.31019278
5	HE	0.4650655	0.58733625	0.61244542	0.61432557	0.65065502	0.70305677
5	HE.G	0.00019651	0.00125	0.00228166	0.00432739	0.00437227	0.01899563
5	HE.GG	0.67663044	0.75475544	0.80434783	0.79257246	0.83016304	1
5	Total.HE	0.00193691	0.00294889	0.0040044	0.0061405	0.00638745	0.02066399
5	N	0.00785061	0.01358087	0.0273421	0.03937771	0.04435309	0.21903366
5	N.G	0.00519165	0.03464655	0.04656045	0.05422551	0.06868642	0.14700562
5	N.GG	0	0.00732282	0.01600122	0.01727934	0.02127008	0.04497412
5	N.S	0	0.06405423	0.09314034	0.13339743	0.14377905	0.65652251
5	Total.N	0.03042831	0.04212637	0.05572566	0.06136086	0.07094559	0.1436506
6	E	0.05602583	0.06934961	0.09960156	0.15537848	0.18563044	0.36628498
6	EG.1	0.00102671	0.00946545	0.0253616	0.04533957	0.06123572	0.12960835
6	E.S	0.16227754	0.17285636	0.18436915	0.34050896	0.35202175	0.83101999
6	Total.E	0.00288386	0.01035156	0.02572063	0.04610959	0.06147865	0.13011323
6	HE	0.57860262	0.58351528	0.61026201	0.6239083	0.65065502	0.69650655
6	HE.G	0.00232533	0.01670306	0.0468286	0.06948144	0.09960699	0.18194323
6	HE.GG	0.69836957	0.74728261	0.79076087	0.77921196	0.82269022	0.83695652
6	Total.HE	0.00403704	0.01848225	0.0485261	0.07115692	0.10120078	0.18353845
6	N	0.02021978	0.02500468	0.11116552	0.1167206	0.20288144	0.2243316
6	N.G	0.22776496	0.29450844	0.32763671	0.30628782	0.3394161	0.3421129
6	N.GG	0.00344432	0.06151329	0.08991079	0.08372506	0.11212256	0.15163434
6	N.S	0.03506708	0.04497621	0.08567245	0.11705011	0.15774635	0.26178847
6	Total.N	0.24595735	0.29017255	0.32074279	0.30623822	0.33680846	0.33750997

Metabolites from flavanones at final time (day 60)							
**clusters**	**metabolite**	**min**	**q1**	**median**	**mean**	**q3**	**max**
1	E	0.03152955	0.06923265	0.09291303	0.09634079	0.12132176	0.22084675
1	EG.1	0.00023615	0.00653513	0.01564535	0.02979461	0.04154532	0.15414903
1	E.S	0.01615008	0.07075563	0.10242371	0.17247138	0.18332971	0.99474511
1	Total.E	0.00088097	0.0077469	0.01606927	0.03032881	0.04195927	0.15437689
1	HE	0.41921397	0.5720524	0.61790393	0.60116448	0.6430131	0.69432314
1	HE.G	0.00058952	0.00417031	0.00854804	0.03966359	0.05421943	0.18724891
1	HE.GG	0.65217391	0.74048913	0.77173913	0.77043076	0.80706522	0.9076087
1	Total.HE	0.00230688	0.00608277	0.01030479	0.04143639	0.05618124	0.18836984
1	N	0.008223	0.01101001	0.02904509	0.0380343	0.05224954	0.12482263
1	N.G	0.00471361	0.02472383	0.03276074	0.03269795	0.04352996	0.05132738
1	N.GG	0.00168236	0.00798121	0.01592931	0.02715301	0.03555663	0.10831688
1	N.S	0.00501246	0.03201113	0.05577103	0.07230573	0.08964869	0.35147313
1	Total.N	0.00834813	0.03080671	0.03442239	0.03579632	0.04561356	0.05601687
2	E	0.02851516	0.06811885	0.09467454	0.0975914	0.12065675	0.18659059
2	EG.1	0.00122072	0.01866242	0.02832252	0.04882404	0.05187557	0.29511692
2	E.S	0.04491294	0.0827006	0.20985946	0.23944767	0.28539639	0.84777932
2	Total.E	0.00186079	0.01916596	0.02868869	0.0494813	0.05265735	0.29530284
2	HE	0.19432314	0.52401747	0.57423581	0.56652967	0.62008734	0.78820961
2	HE.G	0.00227074	0.03783843	0.06437773	0.12496019	0.1639083	0.44478166
2	HE.GG	0.3451087	0.70652174	0.76358696	0.75255755	0.8451087	0.89402174
2	Total.HE	0.00390646	0.03968487	0.06630105	0.12660382	0.1651487	0.44616372
2	N	0.00945266	0.0201899	0.02989623	0.05472295	0.08796809	0.17487615
2	N.G	0.11948528	0.16169875	0.16638741	0.18290338	0.17558561	0.40880363
2	N.GG	0.00480627	0.0111476	0.01212095	0.0433313	0.05319248	0.20419165
2	N.S	0.0329222	0.06744802	0.08799076	0.14907981	0.11516776	1
2	Total.N	0.12328111	0.16794214	0.17184815	0.18721363	0.1830329	0.3929937
3	E	0	0.02424635	0.04161109	0.04648799	0.05858284	0.14085966
3	EG.1	0.00017546	0.00140932	0.0055164	0.01192185	0.01380514	0.08184267
3	E.S	0.00664627	0.04300715	0.07226067	0.08533723	0.10438079	0.26661449
3	Total.E	0.00069373	0.00181707	0.005818	0.01227776	0.01430316	0.08200741
3	HE	0.59388646	0.65829694	0.67030568	0.68405305	0.69759825	0.92576419
3	HE.G	0.00019651	0.00120633	0.00586245	0.02955362	0.01429585	0.31616812
3	HE.GG	0.74456522	0.82065217	0.83967391	0.84581321	0.86684783	0.95108696
3	Total.HE	0.00186074	0.00311211	0.00762794	0.03124807	0.01606655	0.31839302
3	N	0.01055637	0.01425365	0.02827345	0.03054577	0.03025231	0.16677051
3	N.G	0.00079294	0.01293389	0.01687598	0.01884086	0.02206386	0.04347762
3	N.GG	0.00087001	0.0033559	0.00810737	0.01432902	0.01680831	0.08921452
3	N.S	0.00508104	0.01610356	0.03008286	0.03673553	0.03873111	0.13910039
3	Total.N	0	0.01361025	0.02085941	0.01979035	0.02424724	0.04141946
4	E	0.01941372	0.03706328	0.04766828	0.05407654	0.06542331	0.12618382
4	EG.1	0.00168747	0.00707432	0.01387755	0.02496423	0.02745378	0.1768093
4	E.S	0.00292047	0.07448993	0.10927204	0.12715748	0.15339494	0.37230633

4	Total.E	0.0020772	0.00757002	0.01438854	0.02541269	0.02776059	0.17716407
4	HE	0.55458515	0.63864629	0.66157205	0.67093957	0.69650655	0.95633188
4	HE.G	0.0019869	0.00814411	0.01950873	0.04998274	0.04525655	0.30645197
4	HE.GG	0.70923913	0.78125	0.82065217	0.81670757	0.8451087	0.9076087
4	Total.HE	0.00372148	0.00982056	0.02111013	0.0516956	0.04685579	0.30862142
4	N	0.00752552	0.01630399	0.0310068	0.04535763	0.07157005	0.13449983
4	N.G	0.04517414	0.06569905	0.08351424	0.08474622	0.10212401	0.15505073
4	N.GG	0.00307105	0.00931141	0.01478584	0.01931164	0.02522569	0.06096543
4	N.S	0.00638224	0.02829315	0.04868105	0.12520708	0.10733979	0.97268196
4	Total.N	0.0494582	0.07106983	0.0909652	0.09029527	0.10536038	0.1524084
5	E	0.06598684	0.12257458	0.15153174	0.14937896	0.1681504	0.30656355
5	EG.1	0.00106715	0.02264384	0.03434419	0.11647148	0.07073274	1
5	E.S	0.03234868	0.07308022	0.11540139	0.13951931	0.20704689	0.31404165
5	Total.E	0.00169509	0.02300729	0.03481129	0.11694404	0.07154446	1
5	HE	0.27292576	0.41866812	0.51200873	0.48066126	0.55895197	0.59825328
5	HE.G	0.00228166	0.013125	0.01973799	0.02833749	0.0353357	0.11641921
5	HE.GG	0.38586957	0.48165761	0.66983696	0.6265528	0.70516304	0.9375
5	Total.HE	0.00418938	0.015566	0.02165421	0.0301449	0.03706243	0.11790117
5	N	0.00914713	0.01290143	0.02364753	0.02804621	0.04148361	0.06339522
5	N.G	0.05154496	0.07730457	0.09292774	0.09613155	0.11745312	0.14374988
5	N.GG	0.00347736	0.01100172	0.0266664	0.02442247	0.03323425	0.04814586
5	N.S	0.00238126	0.02391862	0.06721857	0.10529721	0.14320787	0.29999453
5	Total.N	0.06918812	0.08448018	0.09290699	0.09927653	0.11368455	0.15897985
6	E	0.15591739	0.22295907	0.26749379	0.30010145	0.27616265	0.75402494
6	EG.1	0	0.00439472	0.0102344	0.01702682	0.02566702	0.05901618
6	E.S	0.08778376	0.17638263	0.21259397	0.22755122	0.28532394	0.40307785
6	Total.E	0.00057287	0.00523849	0.01049252	0.01772655	0.02630058	0.06008867
6	HE	0.39737991	0.5	0.57860262	0.58806405	0.60262009	1
6	HE.G	0.00572052	0.07158297	0.15456332	0.15617419	0.25431223	0.33991266
6	HE.GG	0.63043478	0.65217391	0.73913044	0.72252416	0.7798913	0.84782609
6	Total.HE	0.00739943	0.07308023	0.15581236	0.15783632	0.25563934	0.34118978
6	N	0.01143531	0.04400879	0.06339522	0.16570333	0.08556011	1
6	N.G	0.13276667	0.19826792	0.23780374	0.23786106	0.2728442	0.3166098
6	N.GG	0.00494551	0.0324181	0.05282014	0.07897114	0.11354133	0.22102927
6	N.S	0.03212548	0.07403908	0.13371496	0.14448898	0.1754823	0.31449541
6	Total.N	0.14070772	0.21205078	0.24570341	0.24445176	0.27857136	0.31696706

Table S6. (Poly)phenol metabolites concentration values and more information about the volunteer 66, who was assigned a cluster by the clustering with no one else.

Caffeic Acid (CA), CA-G Glucuronide (CA-G), CA-Glucuronide-Sulfate (CA-GS), total amount of CA and its derivatives (Total CA), 3,4-Dihydroxyphenylacetic acid (DHPAA), DHPAA-Glucuronide (DHPAA-G), DHPAA-Glucuronide-Sulfate (DHPAA-GS), DHPAA-Di-Sulfate (DHPAA-SS), total amount of DHPAA and its derivatives (Total DHPAA), Trans Ferulic Acid- Glucuronide (TFA-G), Trans Ferulic Acid-Suldate (TFA-S), total amount of TFA and its derivatives (Total TFA), Vanillic Acid (VA), VA-Di-Glucuronide (VA-GG) and VA-Di-Sulfate (VA-SS), VA-Glucuronide-Sulfate (VA-GS), total amount of VA and its derivatives (Total VA). Sucralose (SU)

**Time**	**Initial**	**Final**
**CA**	0.02477876	0.08495575
**CA-G**	0.81331816	0.07968127
**CA-S**	0	0.5513544
**CA-GS**	0.002	0.007
**Total CA**	2.88	1.31
**DHPAA**	0.1541353	0.1804511
**DHPAA-G**	0.08283931	0.08616745
**DHPAA-GG**	0	0
**DHPAA-GS**	0.03022339	0.47963206
**DHPAA-SS**	0.07738095	0.20634921
**Total DHPAA**	0.09661139 NA NA	0.11849830
**TFA-G**	0.02258727	0.06091718
**TFA-S**	0.04708022	0.04917003
**TFS-SS**	0	0
**Total TFA**	0.04837226	0.05167209
**TIFA-S**	0	0.03748869
**VA**	0.076369332	0.008281815
**VA-GG**	0.3700495	0.3254950
**VA-GS**	0.1437247	0.5155196
**VA-SS**	0.02612915	0.10936917
**Total VA**	0.08978564	0.04345161
**Cluster**	6	4
**Sweetener**	SU	
**Sex**	WOMAN	

Figure S1. Zoom on CA-GS clusters analyses results

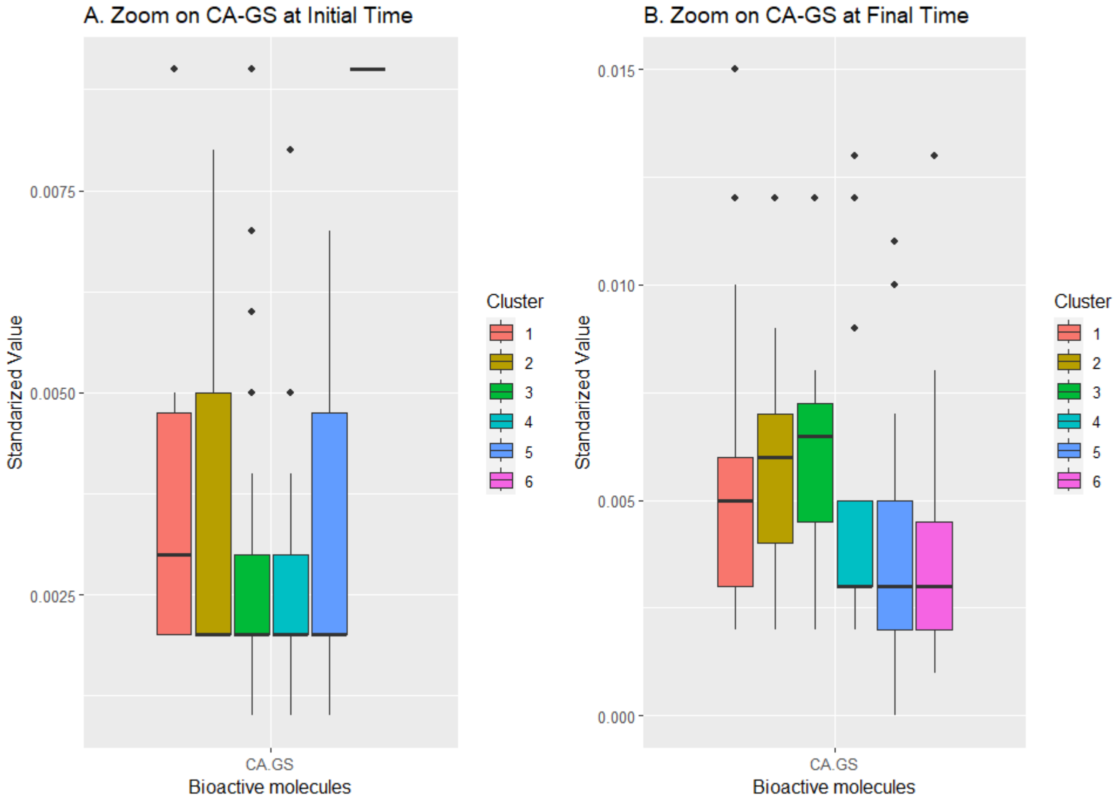

Caffeic acid-Glucuronide-Sulfate (CA-GS)
